# Tropospheric ozone assessment report: Global ozone metrics for climate change, human health, and crop/ecosystem research

**DOI:** 10.1525/elementa.279

**Published:** 2018

**Authors:** Allen S. Lefohn, Christopher S. Malley, Luther Smith, Benjamin Wells, Milan Hazucha, Heather Simon, Vaishali Naik, Gina Mills, Martin G. Schultz, Elena Paoletti, Alessandra De Marco, Xiaobin Xu, Li Zhang, Tao Wang, Howard S. Neufeld, Robert C. Musselman, David Tarasick, Michael Brauer, Zhaozhong Feng, Haoye Tang, Kazuhiko Kobayashi, Pierre Sicard, Sverre Solberg, Giacomo Gerosa

**Affiliations:** * A.S.L. & Associates, Helena. MT, US; † Stockholm Environment Institute, Environment Department, University of York, York, UK; ‡ NERC Centre for Ecology and Hydrology, Penicuik, UK; § School of Chemistry, University of Edinburgh, Edinburgh, UK; ‖ Alion Science and Technology, Inc., Research Triangle Park, NC, US; ¶ Office of Air Quality Planning and Standards, U.S. EPA, Research Triangle Park, NC, US; ** Center for Environmental Medicine, Asthma, and Lung Biology, University of North Carolina, Chapel Hill, NC, US; †† NOAA Geophysical Fluid Dynamics Laboratory, Princeton, NJ, US; ‡‡ NERC Centre for Ecology and Hydrology, Environment Centre Wales, Bangor, UK; §§ Forschungszentrum Jülich GmbH, Jülich, DE; ‖‖ Institute for Sustainable Plant Protection, National Research Council, Florence, IT; ¶¶ Italian National Agency for New Technologies, Energy and Sustainable Economic Development, Rome, IT; *** Key Laboratory for Atmospheric Chemistry, Institute of Atmospheric Composition, Chinese Academy of Meteorological Sciences, Beijing, CN; ††† Department of Civil and Environmental Engineering, The Hong Kong Polytechnic University, Hong Kong, CN; ‡‡‡ Department of Biology, Appalachian State University, Boone,NC, US; §§§ USDA Forest Service, Rocky Mountain Research Station, Fort Collins, CO, US; ‖‖‖ Air Quality Research Division, Environment and Climate Change Canada, Downsview, ON, CA; ¶¶¶ School of Population and Public Health, University of British Columbia, Vancouver, British Columbia, CA; **** Research Center for Eco-Environmental Sciences, Chinese Academy of Sciences, Beijing, CN; †††† Institute of Soil Sciences, Chinese Academy of Sciences, Nanjing, CN; ‡‡‡‡ Graduate School of Agricultural and Life Sciences, The University of Tokyo, Tokyo, JP; §§§§ ACRI-HE, 260 route du Pin Montard BP234, Sophia Antipolis, FR; ‖‖‖‖ Norwegian Institute for Air Research (NILU), Kjeller, NO; ¶¶¶¶ Dipartimento di Matematica e Fisica, Università Cattolica del Sacro Cuore, Brescia, IT

**Keywords:** tropospheric ozone, ground-level ozone, metrics, ozone distributions, shifting ozone concentrations, trends

## Abstract

Assessment of spatial and temporal variation in the impacts of ozone on human health, vegetation, and climate requires appropriate metrics. A key component of the *Tropospheric Ozone Assessment Report (TOAR)* is the consistent calculation of these metrics at thousands of monitoring sites globally. Investigating temporal trends in these metrics required that the same statistical methods be applied across these ozone monitoring sites. The nonparametric Mann-Kendall test (for significant trends) and the Theil-Sen estimator (for estimating the magnitude of trend) were selected to provide robust methods across all sites. This paper provides the scientific underpinnings necessary to better understand the implications of and rationale for selecting a specific TOAR metric for assessing spatial and temporal variation in ozone for a particular impact. The rationale and underlying research evidence that influence the derivation of specific metrics are given. The form of 25 metrics (4 for model-measurement comparison, 5 for characterization of ozone in the free troposphere, 11 for human health impacts, and 5 for vegetation impacts) are described. Finally, this study categorizes health and vegetation exposure metrics based on the extent to which they are determined only by the highest hourly ozone levels, or by a wider range of values. The magnitude of the metrics is influenced by both the distribution of hourly average ozone concentrations at a site location, and the extent to which a particular metric is determined by relatively low, moderate, and high hourly ozone levels. Hence, for the same ozone time series, changes in the distribution of ozone concentrations can result in different changes in the magnitude and direction of trends for different metrics. Thus, dissimilar conclusions about the effect of changes in the drivers of ozone variability (e.g., precursor emissions) on health and vegetation exposure can result from the selection of different metrics.

## 1. Introduction

Tropospheric ozone is a pollutant that is detrimental to human health and crop and ecosystem productivity (REVIHAAP, 2013; US EPA, 2013; Monks et al., 2015; CLRTAP, 2017). Data from widespread observational networks, operational since the 1970s, provide hourly average ozone data from thousands of surface monitoring sites across the globe, and vertical information is available from ozonesondes, aircraft, and satellites (Schultz et al., 2017, hereinafter referred to as *TOAR-Surface Ozone Database*). The data from these networks continue to increase our understanding of ambient ozone levels and their possible impacts on human health, vegetation, and climate change. In addition, this information provides a better understanding about tropospheric ozone distributions, their variability, and long-term changes which are also simulated by global chemistry models (e.g., Fiore et al., 2009; Young et al., 2013). However, uncertainty remains in the spatio-temporal distributions in many regions due to insufficient monitoring (Sofen et al., 2016). Consequently, we rely on global chemistry models to fill gaps in these areas to improve our understanding of long-term changes in tropospheric ozone (Young et al., 2018, hereinafter referred to as *TOAR-Model Performance*).

Since 1990, anthropogenic ozone precursor emissions have decreased in North America and Europe, while increasing in Asia (Granier et al., 2011; Cooper et al., 2014; Zhang et al., 2016). The geographic shift in emissions provides an opportunity to (re)assess the following important questions:

- Which regions of the world have the greatest human and plant exposure to ozone pollution?
- Is ozone continuing to decline in nations with strong emission controls?
- To what extent is ozone increasing in the developing world? and
- How can the atmospheric sciences community facilitate access to ozone metrics necessary for quantifying ozone’s impact on climate, human health, and crop/ecosystem productivity?

To assist in answering these questions, the International Global Atmospheric Chemistry Project (IGAC) developed the *Tropospheric Ozone Assessment Report (TOAR): Global metrics for climate change, human health and crop/ecosystem research* (http://www.igacproject.org/activities/TOAR). Initiated in 2014, TOAR’s mission is to provide the research community with an up-to-date scientific assessment of tropospheric ozone’s global distribution and trends from the surface to the tropopause. TOAR’s primary goals are to: 1) produce the first global tropospheric ozone assessment report based on the peer-reviewed literature and new analyses, and to 2) generate easily accessible, documented data on current ozone exposure and dose metrics as well as trends in these same metrics at thousands of measurement sites around the world (urban and non-urban).

### 1.1. Factors affecting ozone variability

Past assessment of data has shown that over the last several decades, changes in the distribution of hourly ozone concentrations have resulted from (1) the implementation of mitigation strategies aimed at reducing ozone precursor emissions (Gégo et al., 2007; Oltmans et al., 2006, 2013; Kelly et al., 2010; Lefohn et al., 2010a; Wilson et al., 2012; Seguel et al., 2012; Li et al., 2013, 2014; Sicard et al., 2013; Akimoto et al., 2015; Guerreiro et al., 2014; Zhang et al., 2014; Simon et al., 2015; Vedrenne et al., 2015; Lefohn et al., 2017), (2) human activities, which have increased emissions of ozone precursors (Huang et al., 2013; Lee et al. 2014), and (3) changes in meteorology associated with inter-annual variability and possibly climate change, stratosphere-troposphere exchange, and long-range transport (see extensive reviews of Jacob and Winner 2009; Fiore et al., 2015; Monks et al., 2015). Hourly ozone distributions in different locations of the globe will continue to change as a result of further changes in ozone precursor emissions, from further increases in urbanization (Seto et al., 2012), and as a result of changes in climate (von Schneidemesser et al., 2015; Monks et al., 2015). Changes in distributions of ozone concentrations influence the magnitude of specific ozone metrics used to assess spatial and temporal variation in the quantity of ozone relevant for specific impacts (e.g., human health, vegetation, and climate change).

The implementation of emission controls in urban areas, regions, and/or countries worldwide has resulted in a geographically heterogeneous impact on surface ozone levels over Europe and the United States (Sicard et al., 2013; Cooper et al., 2014; Monks et al., 2015; Simon et al., 2015). This is due to the temporal and spatial heterogeneity of emissions changes that have occurred in the past several decades, and to the variability in ozone chemical formation regimes. Emissions of the two major ozone precursors, nitrogen oxides (NO_x_) and volatile organic compounds (VOCs), can have varying impacts on ozone depending on the local conditions. In NO_x_ limited conditions, increases in NO_x_ emissions lead to ozone increases while increases in VOC emissions may have limited impacts (Sillman, 1999). These conditions often occur in locations with lower NO_x_ emission levels (i.e. locations that are rural or downwind of urban plumes and major point sources) and at times of high photochemical activity (i.e., hot sunny summer days) (Sillman, 1999; Murphy et al., 2007; Duncan et al., 2010; Simon et al., 2013). In VOC-or radical-limited conditions, increases in NO_x_ emissions may lead to localized ozone decreases, while increases in VOC emissions result in ozone increases (Sillman, 1999). VOC-limited conditions tend to occur in areas with large NO_x_ emissions (e.g., urban core areas and power plant plumes) and under conditions of lower photochemical activity (e.g., nighttime hours, cloudy days, wintertime days) (Jacob et al., 1995; Sillman, 1999; Murphy et al., 2007; Simon et al., 2013). The NO_x_-limited conditions are conducive to ozone formation and consequently are often associated with times and locations of high ozone (Sillman, 1999; Simon et al., 2013). Conversely, VOC-limited conditions are sometimes, though not always, associated with lower ozone levels (i.e., due to NO_x_ titration near large NO_x_ emissions and/or low photochemical activity in winter or at night). As a result, studies indicate that the large NO_x_ emission reductions that have occurred in the past several decades in the European Union (EU) and US have led to a compression of the ozone distribution, where the high levels shift downward (Butler et al., 2011; Cooper et al., 2014; Cooper et al., 2012; Derwent et al., 2010; Hogrefe et al., 2011; Koumoutsaris and Bey, 2012; Lefohn et al., 2010a; Munir, 2013; Sather et al., 2012; Sicard et al., 2013; Sicard et al., 2016a; Simon et al., 2015; Tripathi et al., 2012; EEA., 2013, 2014a) and the low levels shift upward (Simon et al., 2015; Jenkin et al., 2008; Sicard et al., 2016a). Modeling studies also indicate that decreases in peak ozone are the direct result of large NO_x_ and VOC emissions reductions on both continents (Tagaris et al., 2007; Gilliland et al., 2008; Fiore, 2009; Xing et al., 2015; Jonson et al., 2006; Vautard et al., 2006; Solberg et al., 2005; Derwent et al., 2010; US EPA, 2014a). There is also both modeling (Jonson et al., 2006; Hogrefe et al., 2011; Simon et al., 2013; Downey et al., 2015; Simon et al., 2016; US EPA, 2014a) and observational evidence (Simon et al., 2015; Jenkin et al., 2008; Sicard et al., 2016a) that reductions in the frequency of low levels (i.e., shifts of the lower levels upward) are associated with emissions reductions resulting in less ozone titration by NO.

In addition to changes in local and regional anthropogenic precursor emissions described above, trends in surface hourly ozone distributions can be influenced by other factors. First, ozone may be impacted by changes in meteorology induced by year-to-year variations in weather conditions and by long-term changes associated with climate change. Relationships have been demonstrated between observed surface ozone and individual meteorological variables, such as temperature, humidity, cloud cover, wind speed, surface radiation, boundary layer depth, and boundary layer ventilation and stagnation (Camalier et al., 2007; Oswald et al., 2015; also see extensive reviews of Jacob and Winner 2009; Kirtman et al., 2013; Fiore et al., 2015). Modeling studies also indicate that future climate change may lead to both (1) increases in surface ozone, especially in polluted areas (Kirtman et al., 2013; Fiore et al., 2015), and (2) potentially some decreases in surface ozone levels through enhanced boundary layer ventilation (Trail et al., 2014). Such influences could impose either a climate penalty – an increase in surface ozone in the absence of changes in anthropogenic precursor emissions (Wu et al., 2008) or a climate benefit – a reduction in surface ozone (Trail et al., 2014). In addition, modeling studies suggest that climate-driven changes in stratosphere-troposphere exchange could influence surface ozone at a particular location (e.g., Zeng and Pyle, 2003; Hegglin and Shepherd., 2009). Second, changes in natural ozone precursors and/or their sources (e.g., wetland methane, biogenic VOCs, soil and lightning NO_*x*_, and wild-fires) either from inter-annual meteorological variability, climate change, or land-use change can also influence surface ozone (e.g., Yue et al., 2015; von Schneidemesser et al., 2015). These changes can either shift the entire distribution of hourly ozone (e.g., from methane increases) or can contribute to discrete extreme hourly ozone events (e.g., from wildfires). Third, ozone levels and trends may be impacted by changes in long-range transport. For example, at Mace Head, a site located on the west coast of Ireland, observations of monthly averaged ozone concentrations associated with air masses that had minimal influence from European emissions were noted to have increased significantly between 1987 and 2008, before leveling off and decreasing (Derwent et al., 2013). Similarly, studies have shown that there has been an increase of ozone transported from Asia to the Western US over those last two decades, which appears to have lessened in the past several years (Verstraeten et al., 2015; Lin et al., 2015; Parrish et al., 2017).

### 1.2. Ozone metrics in the context of TOAR

A key aspect of TOAR is to produce an integrated, global assessment of ozone by extending analyses previously undertaken only in specific regions. TOAR has compiled the world’s largest database of ozone observations from sites around the world, and therefore facilitates the comparison of monitoring data on local, regional, national, and international scales. However, as indicated above, there are still many parts of the world which remain undersampled. The database contains several datasets that have been collected for scientific purposes, and TOAR applies globally uniform analyses to measurement series across the world. Most of the metrics described in this paper are available as online service from the TOAR database (Schultz et al., 2017). In TOAR, specific units are used when describing ozone observations and levels of exposure. When referencing an ozone observation, which is measured from moist, ambient air, TOAR follows World Meteorological Organization guidelines (Galbally et al., 2013) and uses the mole fraction of ozone in air, expressed in SI units of nmol mol^–1^. Ozone metrics (e.g., annual 4^th^ highest 8-h daily maximum average ozone value) have typically been developed using the mixing ratio unit of parts per million (ppm) or parts per billion (ppb) which, in the case of ozone, refers to the number of ozone molecules per million or billion moist, ambient air molecules in a fixed volume. In reference to units of nmol mol^–1^ and ppb, Galbally et al. (2013) states: “For all practical purposes the two quantities can be used interchangeably and without distinction”. To maintain consistency with the ozone human health and vegetation research community, TOAR uses units of ppb or ppm (or ppb-hrs or ppb h for cumulative indices) when discussing ozone in terms of an exposure metric. Although the usage of the word “concentration” without specifying atmospheric conditions when referring to mole fraction (nmol mol^–1^) and mixing ratios (ppb) is technically incorrect, the vast amount of literature on ozone health and vegetation effects uses the conventional term “concentration” when referring to an ozone level. This common usage does not distinguish between mixing ratio metrics or true concentrations metrics such as μg m^–3^. To enhance the link to the health and vegetation effects literature and national and international policy, as well as to facilitate the understanding of this paper by health and vegetation effects scientists, the word “concentration” is used when appropriate. Here, we define ‘metrics’ as indices derived from hourly (or higher time resolution) ozone measurements and estimates, which are identified later in the paper to be relevant for assessment of the impacts of ozone on human health, vegetation, model-comparison, or characterization of ozone in the free troposphere. Metrics are calculated by averaging or aggregating ozone data over relevant time periods or as expressed as statistical descriptions of the ozone distribution (see Section 2.3).

The aim of this paper is to provide the necessary scientific background to understand the relevance of and implications for selecting a particular ozone metric to assess spatial and temporal variation in ozone relevant for a particular impact. To achieve this, prior to discussion of the 25 TOAR metrics themselves (4 for model-measurement comparison, 5 for characterization of ozone in the free troposphere, 11 for human health impacts, and 5 for vegetation impacts) in Section 2.3, the basic scientific information (Sections 2.1 and 2.2) underpinning of these metrics is provided. Specifically, we first discuss for human health and vegetation effects the concept of exposure and dose. After introducing these concepts, we describe the scientific evidence, based on controlled experimental studies, empirical observations, and epidemiological research, which provide the background on why specific ranges of ozone levels are associated with individual metrics and why at times the metrics behave differently under changing environmental conditions. In the TOAR effects papers (Fleming et al., 2018 (hereinafter referred to as *TOAR-Health*); Mills et al., 2017 (hereinafter referred to as *TOAR-Vegetation*)), only exposure metrics are applied to characterize present-day ozone observations and trends over time. Data for dose metrics were not available to use by TOAR. Metrics are also specifically defined that can be used to evaluate the ability of global models to reproduce observed patterns of ozone spatio temporal variability.

Varying scientific rationales exist concerning which exposure and dose metrics are most helpful for assessing human health and vegetation effects (e.g., US EPA, 2013, 2014b; REVIHAAP, 2013; CLRTAP, 2017). As a result, in this paper, all exposure and dose metrics are discussed in an equivalent fashion with appropriate clarifications. A suite of metrics needed to evaluate global model results is also described. While we summarize model-measurement comparison metrics in this paper, more details are provided on different approaches for evaluating the models in *TOAR-Model Performance*.

Through the TOAR data portal (http://toar-data.fzjuelich.de/), these ozone metrics are freely accessible for research on the global-scale impact of ozone on climate, human health, and crop/ecosystem productivity. The assessment report is organized as a special issue of *Elementa* (this issue). It is important to note that while the specific ozone-related metrics discussed in this paper relate to TOAR, there exist other metrics used for research and regulatory purposes. Some of these metrics relate to ozone radiative forcing, ozone production efficiency, and “design values” associated with the US EPA’s National Ambient Air Quality Standards.

As a part of the TOAR program, an important consideration is the selection of appropriate statistical tests that can be consistently applied across thousands of measurement sites to quantify changes in distributions and metrics. In Section 3, we discuss some of the statistical approaches available for characterizing trends, as well as the key assumptions associated with these approaches. The rationale by TOAR for selecting the nonparametric Mann-Kendall (M-K) test to identify significant trends and the Theil-Sen (T-S) estimator for estimating the magnitude of the trend is provided.

As described above, controlled experimental studies, empirical observations, and epidemiological research provide the underpinnings that determine the specific ranges of ozone levels associated with the individual metrics. In Section 4, we discuss the response of the various metrics to changes in the distribution of hourly average concentrations, which influence the magnitude of the metric, and the magnitude and direction of change in that part of the distribution. Trends in exposure metrics may change in the same direction as emissions change or may not (Karlsson et al., 2007, 2017; EEA, 2009; Tripathi et al., 2012; Li et al., 2014; Paoletti et al., 2014; Simpson et al., 2014; Malley et al, 2015; Sicard et al., 2016a; Lefohn et al., 2017). The extent to which a human health ozone exposure metric is influenced by low, moderate, or high ozone levels determines whether the metric has decreased, increased, or not changed. A common change in ozone concentration distribution can result in dissimilar trends in health and vegetation metrics because they may differentially emphasize low, moderate, or high ozone levels. It is in fact not uncommon for one metric to show a positive, statistically significant trend, while another shows a negative trend, also significant, for the same ozone time series.

Based on the metrics selected, the results in Section 4 provide a knowledge base from which it is possible to place into perspective the trend results described in *TOAR-Health*, *TOAR-Vegetation*, and Gaudel et al. (2017) (hereinafter referred to as *TOAR-Climate*). Section 4 provides insight into the implications of using specific exposure metrics for assessing potential changes in ozone relevant for human health and vegetation resulting from, or potentially achievable from the implementation of emission control strategies. Distributions and trends are an important aspect of understanding the behavior of exposure metrics as changes occur in emissions, as well as other drivers. It was anticipated that the development of the software and methodology used for quantifying the relationship between changes in distributions of hourly average levels and changes in the magnitude and trend patterns for the various TOAR metrics would be a lengthy process. To maximize the effort, prior to the completion of the TOAR database, a case study was undertaken in which the relationship between changes in the hourly ozone level distributions and a subset (14) of human health and vegetation metrics included in the TOAR database were explored at sites in Europe, the US, and China. The results from the case study (Lefohn et al., 2017) are succinctly summarized in Section 4.1 to introduce the reader to the concepts used throughout Section 4. In Section 4.2, a comparison between trend patterns described in the case study and patterns observed in the metrics using the TOAR database provides evidence that the conclusions from Lefohn et al. (2017) are relevant to the larger set of TOAR metrics. Hence in Section 4, the aim in integrating the results from Lefohn et al. (2017) with expanded analyses using the TOAR database, is to further explore why metrics developed to quantify the same impact (e.g., human health acute effects) provide different estimates of spatial and temporal variation in ozone for a particular impact.

## 2. Exposure and dose metrics

Evidence from different studies on ozone impacts or policy considerations between regions has resulted in a suite of metrics derived from human health and vegetation experiments, as well as developed for model comparison. Data for calculating various metrics may originate from ground-based monitoring networks, and ozonesonde, aircraft, lidar, and remote sensing (including satellite) measurements using different sampling time scales. The official list of TOAR metrics is described at http://www.igacproject.org/activities/TOAR, and a comprehensive list of the statistics calculated in the TOAR database, including the official TOAR metrics, are described in *TOAR-Surface Ozone Database*.

The observed quantification of ozone exposure and dose metrics and its application in human health and vegetation assessments forms the basis for the establishment of legislated air quality standards around the world (SANS, 2011; Kamyotra et al., 2012; dos Santos et al., 2014; McGarity, 2015; Qiao et al., 2015; US Federal Register, 2015; CLRTAP, 2017), and has facilitated regional cooperation in characterizing the transboundary ozone impacts, especially between EU Member States (European Council Directive 2008/50/EC; de Leeuw and Ruyssenaars, 2011) and between the signatories of the UN Convention on Long-range Transboundary Air Pollution (CLRTAP, 2017). These standards provide a legal basis for requiring emissions reductions in areas where human health and vegetation are at risk (AQEG, 2009; EEA, 2014b; Vedrenne et al., 2015; US Federal Register, 2015). The calculation of exposure and dose metrics from hourly averaged ozone measurements across a measurement network provides a consistent method to assess the relative severity of the potential impact to human health or vegetation (US EPA, 2017; Gauss et al., 2014; Guerreiro et al., 2014).

The information in this section provides the (1) definition of exposure and dose, (2) scientific evidence based on controlled experimental studies and empirical observations for focusing on specific ranges of ozone levels for developing exposure and dose metrics, and (3) description and rationale for each metric, including how changes in a specific metric are linked to changes in the ozone concentration distribution. It is important to note that the TOAR database focuses on exposure metrics and leaves the calculation and application of dose metrics to others. Additional information on metrics is provided in Supplemental Material.

### 2.1. Definitions of exposure and dose

For both humans and vegetation, exposure can be defined as the ozone level near the person/plant over time. In some cases, exposure can be defined more specifically by ozone concentration multiplied by time. Dose, on the other hand, refers to the amount of ozone inhaled or absorbed. The next two sections describe how exposure and dose are applied for human health and vegetation.

#### 2.1.1. Human studies

Human health responses are influenced by ozone concentration, duration of exposure, the rate of change of ozone concentration over a period of exposure, frequency of exposures, level of exertion during exposure, health, age, sex, and other risk factors (US EPA, 2013). Lung function and airway inflammation variables are the most frequently used measures to assess the effects of ozone exposure. The development and intensity of typical subjective symptoms, such as cough, shortness of breath, chest tightness, and throat irritation depend on the level of ozone exposure. Human laboratory studies frequently use the product of ozone concentration, duration of exposure, and minute ventilation (the amount of air inhaled or exhaled in one minute) as determinants of effective dose (Silverman et al., 1976). These authors already recognized that “for a given effective dose, exposure to a high concentration for a short period had more effect than a longer exposure to a lower concentration” stating indirectly that peak concentrations induce greater decrements in spiro-metric lung function effects. Minute ventilation, a product of breathing frequency and tidal volume (amount of air inhaled or exhaled in a single breath), reflects the intensity of physical activity. However, other dose metrics (e.g., impact, local, etc.) have been used to express exposure burden on the individual. In general, the health effects response of individuals to ozone inhalation are also influenced by demographic, physiological, exposure, environmental, and socio-economic factors with exposure and physiological factors being the main determinants of the magnitude of exposure-induced health effects. All these factors contribute to considerable inter-individual variability in health response to ozone, which is measured by a variety of physiological tests assessing inflammatory, immune, and symptomatic effects, as well as functional responses primarily of the cardiopulmonary system. Depending on the combination of the above factors during exposure, the response will vary in intensity from minimal respiratory function changes to clinically significant pathophysiological responses of the cardiopulmonary system. Sequential exposures will lead to attenuation of response in many health variables (Folinsbee et al., 1980; Hazucha, 1993). Co-exposure with or a sequential exposure to other air pollutants may have additive, synergistic, potentiating, or antagonistic effects on the extent of physiologic response as compared to the effects of ozone alone (Linn et al., 1994; Hazucha et al., 1994). The findings of human laboratory studies that control most of the abovementioned factors and determinants of health effects response serve as a database for development of population exposure models. Depending on the objectives of the studies, the human health response may be assessed in terms of exposure-response, concentration-response, or dose-response relationships.

Epidemiologic studies generally use ambient concentrations as surrogates for exposure, and the health outcomes of epidemiological studies are assessed on a population scale (REVIHAAP, 2013; US EPA, 2013). Frequently used short-term exposure metrics are 1-h daily maxima, 8-h daily maxima, and 24-h average concentrations (Katsouyanni et al., 2009; Heroux et al., 2015). For long-term studies, seasonal (e.g., April–September) and annual averages of the above metrics have been used (Jerrett et al., 2009; Turner et al., 2016). Both time-series and cohort studies have shown positive associations between exposure to ozone and respiratory health outcomes (REVIHAAP, 2013). However, the strength of association between various exposure metrics, dosimetry, and health response is influenced by the same factors as the acute short-term laboratory studies. In addition, multiple confounding factors, such as temporal and spatial variation in ozone concentration, diverse environmental conditions in various locations and microenvironments, and the prevalence of other risk factors for the health outcome under study may substantially modify the relationship between ozone exposure and the particular health outcome. These factors are in many cases controlled for in the epidemiological models used to derive such associations. The epidemio-logic studies also incorporate lag days into their structure to assess potential health outcomes since specific health outcomes need a certain period of time to develop. Similar to short-term laboratory studies, exposure models may be useful in assessing the overall ozone burden and the severity of health outcomes in a population.

#### 2.1.2. Vegetation

For assessing the potential for ozone to affect vegetation injury, growth and/or yield, exposure is defined as the integral of the instantaneous level over the period the vegetation is exposed to ozone (commonly expressed in unit of mol m^–3^ h or ppm-hrs) (Musselman et al., 2006). Examples of exposure indices are the W126 and AOT40 metrics (see Section 2.3.4). Although not necessarily considered exposure, seasonal average levels (e.g., 12-h daily average values averaged over a specified period) have also been referred to as exposure indices (US EPA, 2013). In contrast, the ozone dose is determined by first calculating the stomatal flux, which is a temporally dynamic measure of the rate of entry of ozone into the leaf (nmol m^–2^ s^–1^). Dose is the total amount of ozone that is absorbed into the leaf through the stomata, in units of nmol m^–2^, over a period of time and is calculated by integrating over time the instantaneous stomatal flux (Fowler and Cape, 1982; Mills et al., 2011b). The flux is accumulated over a species-specific phenological time window and the vegetation-damaging ozone flux is expressed as the Phytotoxic Ozone Dose (POD_Y_), where Y represents a detoxification threshold below which it is assumed that any ozone molecule absorbed by the leaf will be detoxified (Mills et al., 2011b).

### 2.2. Controlled experimental and empirical evidence for focusing on specific ranges of ozone levels for developing exposure and dose metrics

The magnitude of an exposure or dose metric may be impacted by a combination of high, moderate, or low concentrations. In this section, we discuss the evidence for specific concentration ranges within the distribution that are important for human health and vegetation. The specific form of metrics used to assess human health, and vegetation effects vary between regions and countries. Studies which investigate human health or vegetation impacts can reach different conclusions on the nature of exposure- or dose-response relationships because of different biological endpoints and processes. As a result, the metrics used for assessing human health and vegetation impacts provide varying degrees of weighting on the absolute values of the hourly average ozone concentrations that are related to exposure and dose (see description of individual metrics in Section 2.3). For both human health and vegetation, in some cases, there have been attempts to identify concentrations, exposures, and doses, below which no effects are observed (WHO, 2006; de Leeuw and Ruyssenaars, 2011; US EPA, 2013; US Federal Register, 2015; CLRTAP, 2017). There is no consistent evidence of a human health population cutoff for ozone below which no effect is measurable. Other approaches have also been used, including the use of a concentration weighting scheme (e.g., sigmoidal weighting), for assessing potential cumulative vegetation and human health impacts (Lefohn and Runeckles, 1987; Lefohn et al., 1988, 2010b; McDonnell et al., 2010, 2012).

#### 2.2.1. Human studies

Clinical laboratory studies of healthy volunteers, as well as those with pulmonary disease exposed to a wide range of ozone concentrations under a variety of experimental conditions, overwhelmingly employed a square-wave (i.e., constant exposure) ozone concentration profile. The main reason was simplicity of maintaining the exposure chamber atmosphere. However, as the atmospheric data across different regions of the world unequivocally show, at most sites a dominant daily ozone concentration profile varies from hour to hour and is not constant. Relatively few human laboratory studies have compared the pulmonary function and other endpoints response between the square-wave and more realistic exposure profiles. All such studies have been performed in the US.

Controlled human exposure studies that explore induced decrements in lung function indicate that the higher ozone concentrations should carry greater weight than the moderate and lower concentrations (Hazucha and Lefohn, 2007; Lefohn et al., 2010b). Such studies vary the (1) intensity, duration and frequency of exercise from light to very heavy load on a treadmill or a bicycle ergometer to increased minute ventilation, (2) duration of exposures over 6.6-h and 8-h periods, and (3) application of varying hour-by-hour concentrations versus constant concentrations. In the 1980s and early 1990s, US EPA investigators published the initial studies on the effects of 6.6-h exposures on healthy humans (Folinsbee et al., 1988; Horstman et al., 1990). In 1992, the first 8-h exposure study of ozone on lung function comparing the results using a constant concentration and variable concentration profile that mimicked typical diurnal patterns existing under ambient conditions was published (Hazucha et al., 1992). Both the constant and the variable concentration regimes used the same effective dose although the variable regime included exposure to high hourly average ozone concentrations. Compared to the square-wave exposure profile, the hourly lung function decrements in pulmonary function of subjects exposed to the variable concentration regime were substantially greater one hour after the peak exposure, with the conclusion that the higher concentrations should be weighted more than the mid-and low-level values. Several later studies (Adams 2003, 2006a, 2006b) employing either variable (continually changing) or stepwise (increasing or decreasing from one hour to the next) exposure profiles confirmed the results reported by Hazucha et al. (1992). These studies showed that equivalent doses (varying versus constant exposures) produced different responses which depended on the applied hourly ozone concentration pattern.

In contrast to the controlled human exposure study results, which indicate health impacts (lung function decrements in healthy adults) associated with the higher ozone concentrations, epidemiological results appear to indicate that a wider range of hourly average concentrations are important for assessing effects of ozone on premature mortality and morbidity. Bell and Dominici (2008) were unable to identify an ozone concentration below which no effects were observed for the association between short-term ozone exposure and mortality across 98 US communities. However, there is inconsistent epidemiological evidence on whether all hourly average concentrations play an equally important role in assessing epidemiological human health risks for short-term ozone exposure. Stylianou and Nicolich (2009) reported that no association was evident with mortality for values varying between below 10 and below 45 ppb based on analyses conducted on data from 9 US cities. In addition, no association with mortality was observed below specific concentrations in several other epidemiological studies (e.g., Gryparis et al., 2004; Pattenden et al., 2010). In the most recent analysis of the American Cancer Society Cancer Prevention Study-II cohort, a threshold model with a cutoff at 35 ppb marginally improved association between long-term (i.e., annual daily max 8-h) ozone and respiratory mortality. In its decision to change the human health US National Ambient Air Quality Standard (NAAQS) for ozone from 75 ppb to 70 ppb, the US EPA expressed its uncertainty concerning the public health implications associated with changes in relatively low ambient ozone concentrations compared to the higher concentrations (US Federal Register, 2015). The US EPA, while concluding that reducing the highest ambient ozone concentrations would result in substantial improvements in public health, including reducing the risk of ozone-associated mortality, noted that important uncertainties existed in its epidemiology-based risk estimates (US EPA, 2013). These uncertainties were associated with the heterogeneity in effect estimates between locations, the potential for exposure measurement errors, and uncertainty in the interpretation of the shape of concentration-response functions at lower ozone levels (i.e., equivalent to below 20 ppb) (US EPA, 2013; US Federal Register, 2015).

#### 2.2.2. Vegetation

As discussed for human health effects, similar variations in the relative importance of averaging times and high versus mid- and low-level values exist for vegetation metrics. High ozone levels are an important factor when examining exposure indices and plant injury (Heck et al., 1966; Stan and Schicker, 1982). Controlled fumigation experimental results provide some of the evidence for emphasizing the importance of the higher concentrations in comparison to the mid- and low-level values (e.g., US EPA, 1986, 1992, 1996, 2013; Musselman et al., 1983, 1986, 1994; Hogsett et al., 1985; Nussbaum et al., 1995; Yun and Laurence, 1999; Lee and Hogsett, 1999; Oksanen and Holopaninen, 2001; Köllner and Krause, 2003). Using data from controlled experimental studies, evidence exists that cumulative exposure metrics that weight the higher concentrations more than the mid- and low-level values improve the explanatory power over seasonal (i.e., long term) mean metrics in predicting vegetation yield or growth (Lee et al., 1987, 1988; Lefohn et al., 1988; Musselman et al., 1988; Tingey et al., 1989; US EPA, 1996, 2013). However, this is not always the case for some vegetation (e.g., Hayes et al., 2010). In reviewing the existing literature on vegetation effects based on (1) controlled vegetation effects experiments and (2) empirical observations, the US EPA (US EPA, 2013; US Federal Register, 2015) concluded that (1) ozone effects in plants are cumulative, (2) higher ozone concentrations appear to be more important than lower concentrations in eliciting a response, (3) plant sensitivity to ozone varies with time of day and plant developmental stage, and (4) quantifying exposure with indices that accumulate hourly ozone concentrations and preferentially weight the higher concentrations improves the explanatory power of exposure/response models for growth and yield, over using indices based on mean and peak exposure values.

As indicated above, the US EPA based its recommendation on both controlled vegetation effects experiments and empirical observations. A key empirical observation was a multi-year field study conducted at the San Bernardino National Forest in southern California. In the study, forest health improvements were noted because of substantial reductions of the higher hourly averaged ozone levels. The frequency of mid-level concentrations did not substantially change (Lee et al., 2003; Musselman et al., 2006). There was a slow increase in the number of “mid-range” levels from 1980 to 1986, which corresponded to the period following implementation of the US ozone air quality standard. Because of its evaluation, the US EPA (US EPA, 2013; US Federal Register, 2015) recommended exposure indices that (1) accumulate and (2) weight higher hourly average levels more than the “mid-level” values for protecting vegetation from ozone exposure. The US EPA indicated that these exposure indices offered the most appropriate approach for use in developing response functions and comparing studies of ozone effects on vegetation. As part of its rulemaking review process, the US EPA (US EPA, 2013; US Federal Register, 2015) evaluated the use of flux-based indices (described below) and concluded at the time that the approach was less viable than utilizing exposure metrics. The Agency indicated that further research was required to clarify the temporal pattern of detoxification capacity; detoxification did not necessarily follow the same temporal pattern as stomatal conductance (Heath et al., 2009).

Flux-based metrics have been developed in Europe to quantify the accumulation of damaging ozone taken up by vegetation through the leaf stomatal pores over a specified time during daylight hours (Emberson et al., 2000); 21 flux-based critical levels for different responses have been established (Mills et al., 2011b; CLRTAP, 2017). The magnitude of a flux-based metric is dependent not only on ozone concentration variation, but also on the variation in the meteorological and plant conditions (e.g. phenology, soil moisture, temperature, light) that determine the stomatal conductance, thereby controlling the amount of ozone uptake (CLRTAP, 2017). The metric includes the partial closing effect of higher levels of ozone on stomatal conductance (Wittig et al., 2007; Li et al., 2017; Hoshika et al., 2012, 2015) but does not as yet include sluggish stomatal responses, characterized by delays to fluctuating environmental stimuli after exposure to ozone, that have been found in some species (Paoletti and Grulke, 2010; Mills et al., 2016; McLaughlin et al., 2007a). Further research is needed about the impacts of stomatal sluggishness on ozone uptake. For specific conditions, such as drought (Karlsson et al., 2007; Gao et al., 2017), a flux-based metric may accumulate less ozone, even during periods with high hourly ozone levels because plant stomata are partly closed to conserve water.

Flux-based indices have been shown to better represent the spatial pattern of ozone effects on vegetation across Europe, as compared to the exposure-based AOT40 metric (Mills et al., 2011a). Studies have shown that in locations in northern Europe, flux-based metrics can accumulate more ozone during moderate exposures if plant and soil conditions are conducive to ozone uptake than during periods of higher levels that coincide with hot, dry conditions (Karlsson et al., 2007; Malley et al., 2015). Grantz (2014) showed that variation in ozone flux explained a substantially greater proportion of variability (82%) in the effective flux (flux adjusted for diel variation in plant sensitivity to ozone) for Pima cotton compared to variation in ozone level (43%). Flux-based metrics involve accumulation above a fixed flux threshold which is included to represent the detoxification capacity of the plant that varies with vegetation type/species (Mills et al., 2011b). While it is recognized that detoxification should ideally be represented as a dynamic variable rather than as a fixed threshold, modeling approaches are not yet able to take this dynamic variation into account for exposure-based (e.g., AOT40 or W126) or flux-based metrics. Results reported by Wang et al. (2015) for the diurnal changes of ascorbate, a major detoxification agent in the apoplast and leaf tissues of winter wheat, provide evidence for the dynamic nature of detoxification.

Since the 1950s, ozone injury to vegetation has been investigated by plant pathologists using an epidemio-logical approach. They have used a range of metrics from which they focus on different parts of the ozone concentration distribution to quantify injury and damage effects; these different metrics provide varying relationships between exposure/dose and effects (US EPA, 2013). Epidemiological studies of vegetation have mostly used exposure-based metrics, which center on different parts of the concentration distribution, for deriving information on ozone impacts on vegetation under field conditions (Arbaugh et al., 1998; Karlsson et al., 2006; Fishman et al., 2010). As ozone levels typically increase in tandem with increasing water stress (Matyssek et al., 2007), these studies require sophisticated statistical approaches for separating the impacts of ozone from those of co-occurring factors (e.g., Braun et al., 2007; McLaughlin et al., 2007a, b). Several studies have also used stomatal flux, which incorporates the effects of environmental variables on the uptake of ozone by the leaves (e.g., Braun et al., 2014; De Marco et al., 2015; Sicard et al., 2016b). Based on stomatal flux, epidemiologically-based critical levels could be considered for the protection of wheat yield (De Marco et al., 2010) or visible ozone foliar injury on forest trees (Sicard et al., 2016b), although this approach has not been adopted by CLRTAP (2017). Furthermore, plant epidemiology has been used to test/validate other metrics (Baumgarten et al., 2009). For instance, the US 2008 ozone standard explained wheat yield decline better than AOT40-based EU standards (see Section 2.3.4) (De Marco et al., 2010), although the US standard (i.e., 75 ppb) protected fewer sites than the EU standards. Plant epidemiological studies of deciduous tree growth in Switzerland also correlated ozone flux with decreases in stem and shoot growth, with a critical level comparable to that derived above from exposure experiments (Braun et al., 2007, 2010).

### 2.3. Description and rationale for the TOAR exposure and dose metrics

A summary of the TOAR metrics is provided in Table 1. The table provides references to examples of how a specific metric has been used. The description and rationale for the TOAR exposure and dose metrics used for human health and vegetation characterizations are described in detail in Supplemental Material. An additional key component of TOAR is the assessment of modeled ozone levels, and spatial and temporal variability in ozone levels in the free troposphere from surface, remote sensing, and aircraft-based instruments. These topics are comprehensively discussed in the *TOAR-Climate* and *TOAR-Model Performance* papers for free tropospheric ozone, and modeled ozone levels, respectively. Supplemental Material also includes descriptions of those metrics used for global model-measurement comparison, and for free tropospheric ozone characterizations. This section provides a condensed description of the widely-used ozone metrics for assessing impacts associated with human health, vegetation, and climate change, including their focus on different parts of the distribution of hourly average ozone concentrations.

#### 2.3.1. Model-measurement comparison metrics

Observational metrics calculated at individual sites can provide insight into the physical and chemical processes that determine ozone and its variations on different timescales (e.g., Logan, 1985; Oltmans and Levy II, 1994). Hence, comparison of these metrics calculated at surface sites with modeled ozone levels is one method used to evaluate the performance of global models in predicting tropospheric ozone. Besides uncertainties in observations, a major problem in the comparison of site-specific data with model output is the representativeness of the available measurements. Problems related to the comparison of spatially and temporally sparse observations with coarse resolution global scale models, discussed in more detail in *TOAR-Model Performance*, can be mitigated by comparing model output against globally gridded observational data products that have been aggregated based on site characterization (e.g., *TOAR-Surface Ozone Database*). Table 1 summarizes the TOAR metrics (http://www.igacproject.org/activities/TOAR) used for model-measurement comparisons (based on hourly average levels), which are:

- The monthly mean of the 24-h average (MMEAN) (in units of ppb);
- The monthly standard deviation, median, 5^th^, 25^th^, 75^th^, and 95^th^ percentiles of the maximum daily average 8-h (MDA8) ozone values (in units of ppb);
- The monthly mean diurnal cycle (monthly average of 1-h ozone averages at 0100 h, 0200 h, 0300 h, etc.) (in units of ppb); and
- Monthly mean of daily minimum and maximum hourly average ozone (in units of ppb).

The MMEAN ozone at individual sites is commonly used to study surface ozone variability for global model-measurement comparisons (see *TOAR-Model Performance*). The magnitude of MMEAN depends upon the influence of precursor emissions, photochemistry, meteorology, and atmospheric transport on the shape of the annual cycle of ozone at individual sites. Comparison of simulated and observed MMEAN provides a first order estimate of the model’s ability to simulate the observed annual cycle as well as long-term trends and inter-annual variability. However, the MMEAN smooths the pronounced diurnal cycle observed at continental rural sites due to photochemical ozone production and/or enhanced nighttime surface ozone deposition or in-situ chemical loss under shallow nocturnal boundary layers. Global models at coarse resolution may have difficulty in reproducing these low nighttime values (e.g., Derwent et al., 2004) because of errors in representing the nocturnal boundary layer (Lin et al., 2008) and because many chemical processes are nonlinear and therefore may not be accurately simulated when spatially averaging sharp gradients over larger grid-cells. Therefore, global model evaluation against observed MMEAN at individual sites with strong diurnal cycles should be supplemented with comparison against metrics which characterize the observed diurnal cycle (discussed below) to estimate their ability in reproducing observations (see *TOAR-Model Performance*). The MMEAN exposure metric smooths the large day-to-day variability that occurs at many polluted sites.

The MDA8 exposure metric is an air quality metric used by the US EPA to assess compliance with the NAAQS for ozone to protect human health and vegetation. As a part of the development of the US NAAQS, global chemistry models, using the MDA8 metric, were applied in combination with regional photochemical models to estimate background (US EPA, 2014b) ozone to examine the influence of ozone formed from natural and international sources (e.g., Reidmiller et al., 2009; Zhang et al., 2011; Fiore et al., 2014; Dolwick et al., 2015). Comparison of observed to simulated MDA8 levels provides an assessment of the ability of models to reproduce the trends and variability in this metric used for assessing human health impacts.

The monthly mean diurnal cycle provides information on average daily fluctuations in surface ozone. Diurnal variations in surface ozone are driven by variations in photochemistry, boundary layer dynamics, surface dry deposition, and transport.

The monthly average of daily minimum and maximum of hourly average levels depend on ozone production and loss processes, and transport patterns occurring at a specific site. Comparing modeled and observed diurnal cycles and diurnal ranges is one means by which to evaluate model representation of the many processes that determine the simulated diurnal cycle (e.g., Schnell et al., 2015).

**Figure 1:**
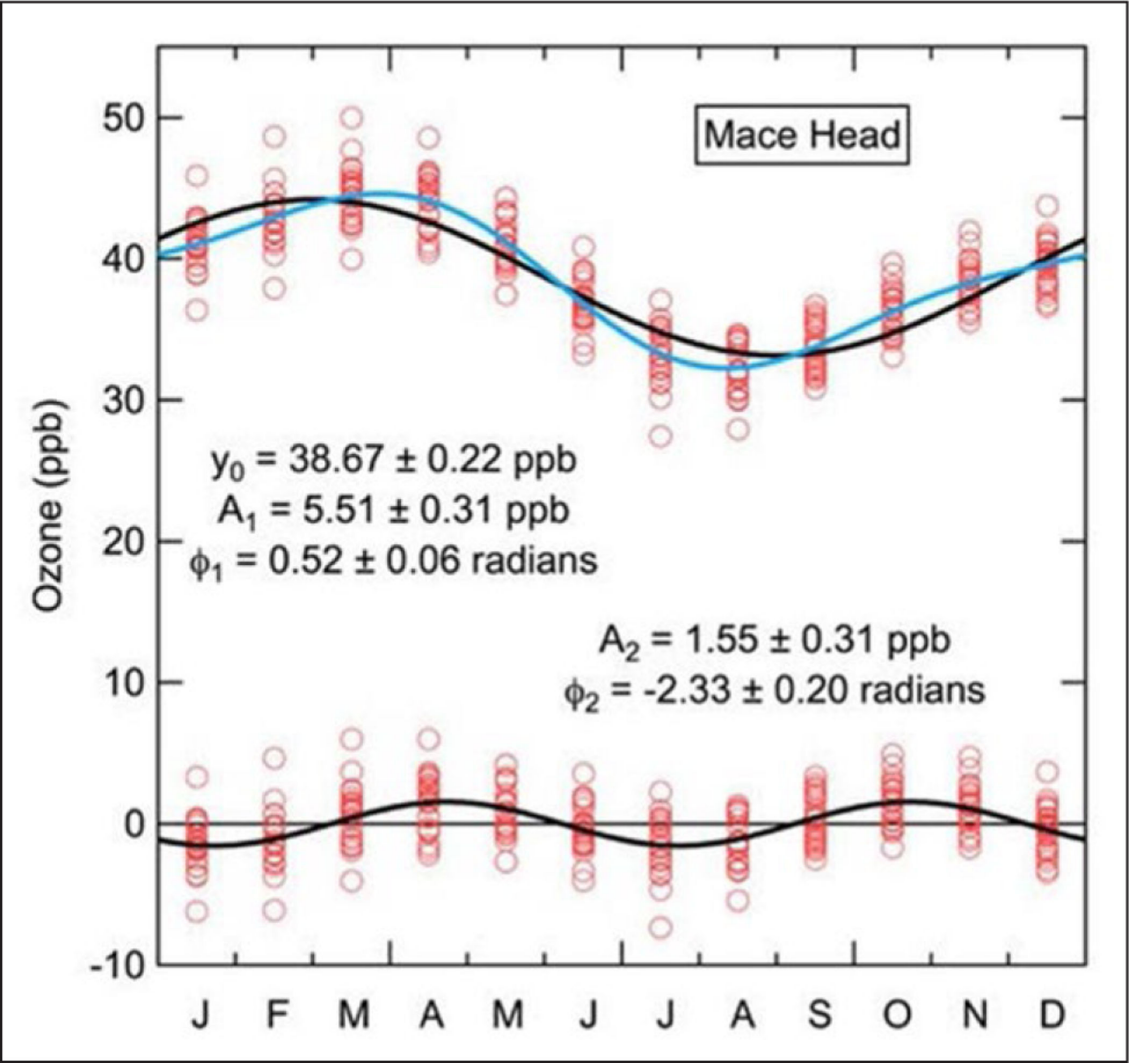
Sine function fits to monthly average data from Mace Head, Ireland. The black curves give the least-squares regressions to the fundamental (upper black curve) and second harmonic (lower black curve) terms, and the blue curve shows their sum. The data points about the x-axis are the residuals between the measurements and the fundamental fit. The fit parameters with 95% confidence limits are annotated. A small, long-term trend has been removed from the monthly average data before fitting (data from Parrish et al., 2016). DOI: https://doi.org/10.1525/elementa.279.f1

In addition to the metrics outlined above, other metrics have been defined, which similarly aim to evaluate the ability of models to represent measured ozone levels. Two alternative sets of metrics have been reported in the literature for global model-measurement comparison specifically related to assessment of long-term changes in baseline ozone (Parrish et al. 2014) and on the seasonal cycle of ozone at marine boundary layer sites (MBL) (Parrish et al. 2016) (See Supplemental Material for a more comprehensive description). The first approach calculated polynomial “shape factors” that define long-term trends of seasonally averaged, baseline ozone levels at relatively remote sites from the mid-20^th^ Century to the present. The metrics produced to compare measured and modeled changes in baseline ozone at northern mid-latitudes are polynomial coefficients, shown in Supplemental Material, Table S-2), which characterize relative (to year 2000) ozone changes over broad regions of northern mid-latitudes. For application of these metrics, see *TOAR-Model Performance*). Secondly, Fourier series expansions of monthly average ozone levels at selected sites provide a series of comparison metrics (Parrish et al., 2016; Derwent et al., 2016). This method represented the seasonal cycle at marine boundary layer sites around the globe as the annual average plus two sine function terms – the fundamental (period = 1 year) and second harmonic (period = 1/2 year). Figure 1 illustrates one example. The parameters from this representation of the seasonal cycle provide metrics which have been shown to provide critical tests of the model treatment of some of the physical processes that control tropospheric ozone levels in the MBL (see *TOAR-Model Performance*).

#### 2.3.2. Free tropospheric metrics

Multiple sources of data (e.g., ozonesonde, aircraft, lidar, and remote sensing) are used to assess ozone throughout the depth of the troposphere as part of the TOAR project. The purpose of free tropospheric metrics (Table 1) is to characterize temporal and longitudinal, latitudinal, and altitudinal spatial variability in ozone levels throughout the troposphere and provide additional insight into the physical and chemical processes occurring that may affect surface ozone. The metrics associated with characterizing the free troposphere are:

- Monthly, seasonal, annual and decadal means from ozonesonde, aircraft, and lidar measurements on pressure surfaces at intervals of 25 hPa from 1000 hPa to the tropopause. Standard deviations, median and 5 th, 25^th^, 75^th^, and 95^th^ percentiles are provided where sampling is sufficient. (Units are ppb)
- Monthly mean diurnal cycle at hourly intervals with high frequency aircraft data (MOZAIC-IAGOS), and lidar where data frequency permits. (Units are ppb)
- Monthly mean tropospheric ozone column (TCO) in Dobson Units (DU) from satellite instruments (OMI/MLS, IASI, GOME, SCIAMACHY, TES) harmonized to a common horizontal grid (e.g., 1° *×* 1.25° as for OMI/MLS). A common tropopause definition is preferred but in any case, the tropopause definition must be specified (e.g., WMO, 1992; Tuck et al., 1985). For instruments with more than one degree of freedom in the troposphere, upper and lower tropospheric integrals, also in DU, are supplied.
- Monthly mean (Total Column Ozone (TCO)) from ozonesondes: the integral in DU of ozone from the surface to the thermal tropopause (WMO, 1966).
- Estimates of the annual cycle, at monthly intervals, averaged over each decade on 25 hPa pressure surfaces or for TCO. Decades defined as e.g. 1960–1969 inclusive. (Units are ppb/Dobson)

These metrics are intended for use in global chemical transport and climate model evaluation, trend analyses, climate studies, and studies of large-scale processes, such as long-range transport, stratosphere-troposphere exchange, and biomass burning. Note that while global model evaluations often compare metrics such as mean or ozone percentiles, regional photochemical model evaluations generally focus on whether ozone was predicted accurately at the right time and location and thus regional model evaluations look at bias and error in paired hourly or daily ozone levels matched in space and time (Simon et al., 2012). However, this straightforward approach may not fairly evaluate model skill, as modest forecast errors in, say, the time or location of an ozone plume may contribute excessively to the total statistical error, as the forecast is too low where the plume should be, and too high where the model placed it (Tarasick et al., 2007). The metrics described in this section aim to provide a general and versatile statistical description of the free tropospheric ozone field, from available measurement sources. All ozone values are in nmol mol^–1^, except for the integrated TCO values, which are given in Dobson units (DU). Because the frequency of observations varies over a large range (e.g., from typically 3–4 per month for ozonesonde data to as frequent as daily profiles during campaigns, or multiple daily profiles by commercial aircraft over some airports), the number of observations in each data sample is also provided to allow averages to be weighted, and/or evaluated for representativeness.

#### 2.3.3. Human health exposure metrics

Exposure metrics used for assessing the potential impacts of ozone on human health focus on different parts of the distribution of hourly average concentrations. Some of the metrics focus on the relatively higher ozone values, while other metrics focus on a combination of the various parts of the distribution. Supplemental Material describes the exposure metrics in detail. Table 1 lists the various TOAR human-health exposure metrics (http://www.igacproject.org/activities/TOAR).

##### 2.3.3.1. Exposure metrics that focus on higher ozone concentrations

The following metrics are influenced by ozone concentrations at the high end of the distribution and have been used for assessing ozone relevant for human health:

- The 4^th^ highest MDA8 ozone value (in units of ppb) over the entire year.
- The maximum daily 1-h average ozone value (in units of ppb) over the entire year.
- The number of exceedances of daily maximum 1-h values greater than 90, 100, and 120 ppb.
- The 4^th^ highest W90 5-h cumulative exposure index (ppb-hrs) as described in Lefohn et al. (2010b). and
- The running mean of the 3-month average of the daily 1-h maximum (in units of ppb) is a metric that is based on epidemiology results.

Several exposure metrics have been defined which are associated with the 4^th^ highest MDA8 concentrations. See Supplemental Material for additional details on various ways to calculate the 4^th^ highest MDA8.

EU information thresholds have been established as hourly ozone concentrations ‘beyond which there is a risk to human health from brief exposure for particularly sensitive sections of the population’, and about which the public must be informed (European Council Directive 2008/50/EC). The following maximum daily 1-h average exposure index focuses on the higher values and is useful for comparing health-relevant ozone at a site with the EU ‘information threshold’, set at 180 μg m^–3^ (90 ppb). The directive also has an alert threshold of 240 μg/m^3^ (120 ppb).

The metrics representing the number of exceedances of daily maximum 1-h values greater than 90, 100, and 120 ppb focus on the high end of the distribution and are used in this assessment as ozone metrics for human health, together with other metrics. In 1979, the US EPA adopted the daily maximum 1-h value of 120 ppb as an air quality standard for ground-level ozone. This 1-h standard was revoked in 2005 by the US EPA, but some areas have continued obligations under this standard (https://www3.epa.gov/ttn/naaqs/criteria.html). The daily maximum 1-h value is still used in some other countries as the ozone standard. For example, Japan has been using the daily maximum 1-h value of 60 ppb as an ozone standard (http://www.env.go.jp/en/air/aq/aq.html). China has established ozone standards using both daily maximum 8-h (75 ppb or 160 μg m^–3^ at 273 K and 101.325 kPa) and daily maximum 1-h (93 ppb or 200 μg m^–3^ at 273 K and 101.325 kPa) metrics for both residential and commercial areas (http://kjs.mep.gov.cn/hjbhbz/bzwb/dqhjbh/dqhjzlbz/201203/t20120302_224165.shtml).

**Figure 2:**
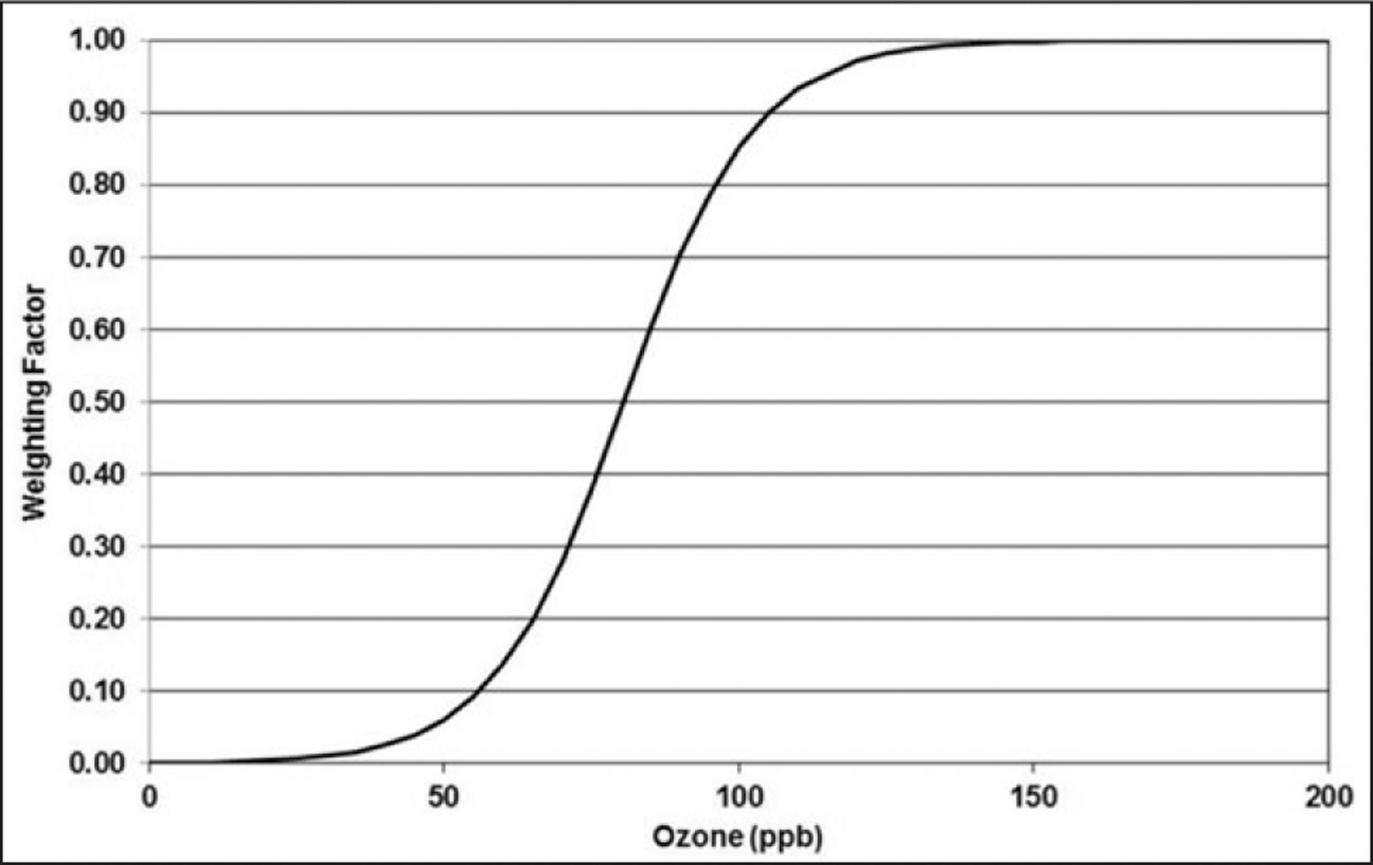
The weighting applied to hourly average ozone values for the calculation of the W90 exposure index (see Lefohn et al., 2010b). DOI: https://doi.org/10.1525/elementa.279.f2

The 4th highest W90 5-h cumulative exposure index is an experimental exposure metric that weights the higher hourly average concentrations more than mid-level values and has been suggested as a relevant human health metric based on controlled human laboratory studies (Section 2.2.1). The rationale for this metric is derived from the analyses summarized in Lefohn et al. (2010b). The form of the W90 index is Ʃ *w*_*i*_ *× C*_*i*_ with weight *w*_*i*_ = 1/[1 + *M × exp* (–*A × C*_*i*_/1000)], where *M* = 1400, *A* = 90, and where *C*_*i*_ is the hourly average ozone mixing ratio in units of ppb. The W90 index has units of ppb-hrs. The weightings for the hourly average values are shown in Figure 2 below.

Finally, the running mean of the 3-month average of the daily 1-h maximum metric is used in TOAR because of its application to estimates of globally deaths attributable to long-term ozone exposure by the Global Burden of Disease project (Forouzanfar et al., 2015; Brauer et al., 2016). The Jerrett et al. (2009) study evaluated the risk of mortality associated with the average of the second (April through June) and third (July through September) annual quarterly averages daily maximum 1-h ozone concentrations. Since the ozone (summer) season varies throughout the globe, the Global Burden of Disease studies used, as the estimate of long-term ozone exposure, the annual maximum of running 3-month average daily maximum 1-h values (Forouzanfar et al., 2015; Brauer et al., 2016). The TOAR long-term trend results (1995–2014) indicate that this human health metric appears to be more associated with the higher hourly concentrations within the distribution than those values associated with the entire distribution (see Section 4). Coupled with this metric, TOAR reports the day of the year on which the 3-month maximum metric reaches its maximum value.

##### 2.3.3.2. Exposure metrics that focus on the high and mid-level ozone concentrations

Exposure metrics that focus on both the high-, as well as at times the mid-level concentrations, are:

- The number of exceedances of daily maximum 8-h values greater than 50, 60, 70, and 80 ppb per year which indicate yearly non-attainment occurrences for some air quality standards used around the globe (e.g., US Federal Register, 2015); and
- The SOMO35 is defined as the annual sum of the positive differences between the daily maximum 8-h ozone average value and the cutoff value set at 35 ppb (70 μg/m^3^) calculated for all days in a year. The unit is ppbday. The 8-h average values are determined as per EU protocols (European Council Directive 2008/50/EC). The metric is consistent with WHO recommendations for quantification of ozone associated with health impacts resulting from short-term exposure (REVIHAAP, 2013). The ozone value selected as the cutoff was chosen partly due to the more accurate modeled ozone values available above 35 ppb, but also due to the observation of a statistically significant increase in mortality calculated for short-term exposure to ozone values above 25–35 ppb (Gryparis et al., 2004).

##### 2.3.3.3. Exposure metrics that focus on high-, mid-, and low-level ozone concentrations

• The SOMO10 metric is the annual sum of the positive differences between the daily maximum 8-h average ozone value and the cutoff value set at 10 ppb (20 μg/m^3^) calculated for all days in a year. The unit is ppb-day. The 8-h average values are determined as per EU protocols (European Council Directive 2008/50/EC). The SOMO10 metric is calculated in the same way as SOMO35, but with the lower cutoff value and reflects the epidemiological evidence of associations between short-term ozone exposure and lower ozone levels (REVIHAAP, 2013).

##### 2.3.3.4. Concentration-based metrics that include ozone concentrations from across the distribution

The following metrics are useful for assessing the distribution of hourly average concentrations:

- The annual and seasonal percentiles (median, 5^th^, 25^th^, 75^th^ and 95^th^) of all hourly average concentrations. Long-term changes in these percentile metrics facilitate the assessment of the impacts of the ozone level associated with different factors. Long-term changes in ozone precursor concentrations can cause trends for different parts of the frequency distribution of ozone concentrations (Lefohn et al., 1998, 2010a; Brönnimann et al., 2002; Xu et al., 2008; Simon et al., 2015), which are not necessarily consistent. Therefore, studying the long-term variations of ozone using these percentile metrics can help to avoid potential misinterpretation in a risk analysis using single summary statistics (e.g., the mean ozone concentration).

##### 2.3.3.5. Epidemiological metrics that focus on chronic exposure

Short-term increases in ozone have been linked to a wide array of health responses, including increases in daily mortality (Thurston and Ito, 2001; Bell et al., 2004; Bell et al., 2007). Studies of the impacts of chronic exposure, which are generally thought to have the greatest population health impact, are less common (REVIHAAP, 2013). Chronic exposure can result from repeated elevated concentrations over time. The following TOAR exposure metric is used for characterizing chronic exposure:

- The annual and summertime mean of the daily maximum 8-h values (in units of ppb) are metrics that were used as an estimate of long-term ozone exposure in an updated epidemiological analysis to the Jerrett et al. (2009) study performed by Turner et al. (2016). Turner et al. (2016) calculated significant association between annual and summertime (i.e., April–September) average daily maximum 8-h ozone values, and all-cause, respiratory, and circulatory mortality within the American Cancer Society Cancer Prevention Study-II (ACS CPS-II) cohort population.

#### 2.3.4. Vegetation metrics

Exposure metrics used in assessing potential impacts on vegetation, similar to human health metrics, focus on different parts of the hourly average concentration distribution. Some of the metrics focus on the relatively higher ozone values, while other metrics focus on a combination of the various parts of the distribution. The Supplemental Material describes the vegetation exposure metrics in detail. Table 1 shows the various TOAR vegetation metrics (http://www.igacproject.org/activities/TOAR) and the parts of the concentration distribution on which they focus. The vegetation metrics are defined by growing season and climate zones (*TOAR-Vegetation*; http://www.igacproject.org/activities/TOAR).

##### 2.3.4.1. Exposure metrics that weight the higher ozone levels and include mid-level values

**Figure 3:**
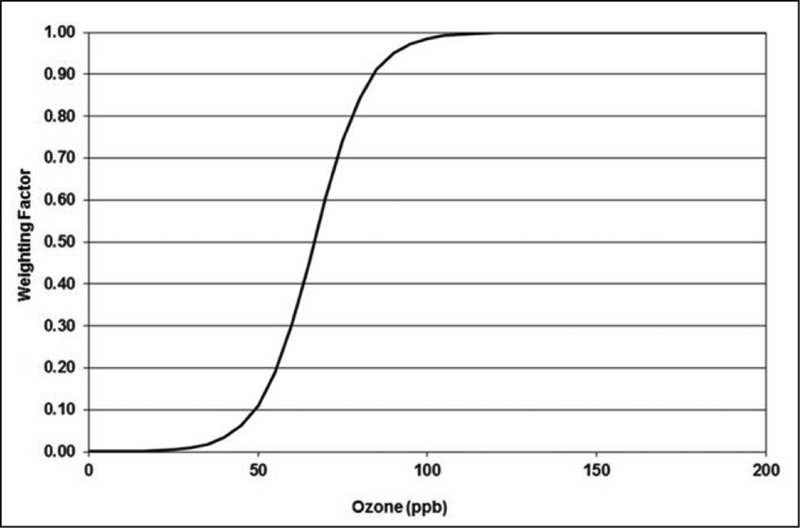
The weighting applied to hourly average ozone values for the calculation of the W126 exposure index (see Lefohn et al., 1988). DOI: https://doi.org/10.1525/elementa.279.f3

The following vegetation exposure metrics focus on the higher levels but include the mid-level values:

- The W126 exposure index (in units of ppb-hrs) is a non-threshold index that is described as the sigmoidally weighted sum of all hourly ozone values observed during a specified daily and seasonal time window, where each hourly ozone value is given a weight that increases from zero to one with increasing value. The W126 metric is identified by the US EPA for assessing risk to vegetation from ozone exposure (US EPA, 2013, 2014a; US Federal Register, 2015). The W126 exposure index has the form: W126 = Ʃ *w*_*i*_ × *C*_*i*_ with weight *w*_*i*_ = 1*/*[1 + *M* × *exp* (*–A* × *C*_*i*_*/*1000)], where *M* = 4403, *A* = 126, and where *C*_*i*_ is the hourly average ozone mixing ratio in units of ppb. Further details about the index are available in Supplemental Material. The weightings for hourly average values are shown in Figure 3. For both this metric, as well as the human health (W90) exposure index mentioned previously, the sigmoidal weightings are similar except at the lower levels (compare Figures 2 and 3), where the W126 provides slightly greater weight than the W90 metric. In the TOAR program, the W126 exposure index is specified over the following time periods: (a) W126 (3-month, 24-h), (b) W126 (6-month, 24-h), (c) W126 (7-month, 24-h), (d) W126 (12-month, 24-h), (e) W126 (3-month, 12-h (0800–1959h) (monthly periods specified), (f) W126 (6-month, 12-h (0800–1959h) (monthly periods specified), (g) W126 (7-month, 12-h (0800–1959h) (monthly periods specified), and (h) W126 (12-month, and 12-h (for tropical or subtropical moist climate zones) (0800–1959h).
- AOT40 is the sum of the difference between the hourly mean ozone value at the top of the canopy and levels above 40 ppb for all daylight hours over a specified time. The unit of the exposure index is ppb h and was originally derived because of a growing understanding that plants responded to accumulated ozone above a threshold rather than a long-term average (Fuhrer et al., 1997). As a threshold, 40 ppb was suggested as being relevant at the time when the contribution of baseline ozone to levels in Europe was thought to be relatively lower than current levels and to clearly separate out the peaks, which are of regional (i.e., European) origin (described in CLRTAP, 2017). In recent years, the CLRTAP has adopted the flux-based metric, POD_Y_ in preference to AOT40 as this metric has greater biological relevance and is better correlated with field evidence of effects (Mills et al., 2011a). The AOT40 is a threshold metric, which at times can be sensitive to small changes near its threshold value (Hollaway et al., 2012). AOT40 is used as the legislative standard in Europe (Directive 2008/50/EC), when accumulated over a standard time window (0800–1959 h) and a standard time period (May to July), although other periods are available in TOAR (Supplemental Material). It is also included in CLRTAP (2017) for daylight hours with vegetation-specific accumulation periods and timings. In the TOAR program, the AOT40 exposure index is specified over the following time periods: (a) AOT40 (3-month, 12-h (0800–1959h), (monthly periods specified according to crop type and growing season and does not apply to forests)), (b) AOT40 (6-month, 12-h (0800–1959h), (monthly periods specified and applicable to perennial vegetation including forests, grassland and perennial crops)), (c) AOT40 (7-month, 12-h (0800–1959h), (monthly periods specified)), (d) AOT40 (12-month, and 12-h (for tropical or subtropical moist climate zones) (0800–1959h)), (e) AOT40 (3-month, daylight over the period when clear sky radiation >50 W m^–2^), (f) AOT40 (6-month, daylight over the period when clear sky radiation >50 W m^–2^), (g) AOT40 (7-month, daylight over the period when clear sky radiation >50 W m^–2^), (h) AOT40 (3-month, nighttime over the period when clear sky radiation <5 W m^–2^), (i) AOT40 (6-month, nighttime over the period when clear sky radiation <5 W m^–2^), and (j) AOT40 (7-month, nighttime over the period when clear sky radiation <5 W m^−2^). The specific steps associated with calculating the AOT40 are provided in Supplemental Material. The threshold for daylight versus night is 5 degrees solar elevation angle, which is used as a surrogate for 50 W m –2 (*TOAR-Surface Ozone Database*).

##### 2.3.4.2. Exposure metric that focus on the mid-range of hourly average ozone levels

The TOAR vegetation exposure metric that is focused on the mid-range of hourly average levels is:

- The daily 12-h (0800–1959h) average exposure metric (M12) (in units of ppb) was widely used in the past to characterize crop exposures to establish crop-specific exposure–response relationships, which relate a quantifiable mean to a reduction in crop yield (Heck et al., 1988; Jäger et al., 1992; Legge et al., 1995). In post-experimental data analysis, cumulative metrics, such as the SUM06 (the accumulation of all hourly average values equal to and above 0.06 ppm) and W126 indices (US EPA, 2013; US Federal Register, 2015) better fit the yield loss observations for experiments conducted in the US, and thus have received greater focus than average metrics (Tingey et al., 1991; Lefohn and Foley, 1993; Mauzerall and Wang, 2001). Supplemental Material provides additional information on the 12-h exposure metric. In the TOAR program, the M12 exposure index is specified over the following time periods: (a) M12 (3-month, 12-h for wheat and rice), (b) M12 (6-month_,_ 12-h), (c) M12 (7-month, 12-h), and (d) M12 (12-month, 12-h for tropical or subtropical moist climate zones) (0800–1959h).

##### 2.3.4.3. Flux-based metric

Currently, flux-based metrics are not characterized in the TOAR database, but are discussed in this section for completeness. The flux-based metric is described as:

- The accumulated Phytotoxic Ozone Dose (i.e., the accumulated stomatal flux) of ozone above a flux threshold of Y (POD_Y_). The POD_Y_ is calculated for the appropriate time-window as the sum over time of the differences between hourly mean values of ozone stomatal flux (*F*_*st*_) and Y nmol m^–2^ PLA s^–1^ for the peri ods when *F*_*st*_ exceeds Y during daylight hours, where PLA is defined as projected leaf area, or, the one-sided area of a leaf perpendicular to the incoming radiation. The DO_3_SE model (Emberson et al., 2000) was adopted by CLRTAP for calculating the accumulated stomatal flux of ozone from hourly values of ozone, together with the following stomatal conductance modifying factors: temperature, vapor pressure deficit (VPD), light (irradiance), soil water potential (SWP) or plant available water (PAW), ozone value, and plant development stage (phenology). The Y threshold varies between species as do the parameterizations for each of the flux modifying factors. Two types of POD_Y_ model exist: POD_Y_IAM which has a simplified parameterization and is suitable for large-scale integrated assessment, and POD_Y_SPEC, species-specific parameterization of the flux model. Local and regional parameterizations have been defined for POD_Y_SPEC and POD_Y_IAM in CLRTAP (2017) for a range of crops, tree and grassland species/species groups and used to define 21 critical levels, above which negative effects of ozone on crop yield, biodiversity and tree growth are expected. Further information can be found in summary in Mills et al. (2011b), and in more detail including response functions in Grünhage et al. (2012) for wheat, Gonzalez et al. (2014) for tomato, and Büker et al. (2015) for tree species. The POD_Y_ model has also been applied in China to derive flux-effect relationships for wheat (Feng et al., 2012) and poplar (Hu et al., 2015).

##### 2.3.4.4. Exposure metrics that include ozone concentrations from across the distribution

For vegetation purposes, the TOAR metrics that focus on the entire distribution are:

- The seasonal (i.e., December–February, March–May, June–August, and September–November) percentiles (median, 5^th^, 25^th^, 75^th^, 95^th^, 98^th^, and 99^th^) of hourly average ozone. The units are ppb. Trends in each percentile by season can provide information on specific changes that occur within the ozone distribution. These changes influence the magnitude of the exposure and dose metrics.

## 3. Statistical and methodological approaches available for TOAR analyses

Trends are defined as whether the data exhibit an overall increase, decrease, or no discernible change over the time period of interest. When testing for trends, normally one first proposes a null hypothesis (here, that there is no trend) and a threshold value, called the significance level of the test, which is traditionally denoted as α. The α value is supposed to be fixed in advance and thus part of the study design, whereas the *p*-value is a number computed from the data and thus, unknown until it is computed. If the *p*-value is less than or equal to the selected significance level (α), this suggests that the observed data are inconsistent with the null hypothesis, and the null hypothesis is rejected.

When the null hypothesis is not rejected, this does not prove that it is true. When the *p*-value is calculated correctly, this test guarantees that the Type I error rate is at most α. A Type I error (sometimes called a “false positive”) occurs when in fact the null hypothesis is true, but one declares that the data are not compatible with it. The *p*-value resulting from the test provides a quantification of making this type of error. For the TOAR trends analysis, a standard α = 0.05 cutoff has been selected (i.e., the null hypothesis is rejected when *p* ≤ 0.05 and not rejected when *p* > 0.05). By itself, the *p*-value does not support reasoning about the verisimilitude of the hypotheses; it is merely a tool for deciding whether to reject the null hypothesis.

A second type of error one may make in hypothesis testing is a Type II error (sometimes called a “false negative”). This occurs when the null hypothesis is in fact not true, but one fails to detect this. A Type II error may result for various reasons, and one may wish to collect more data and/or further examine the existing data in more detail in future research investigations. The ability to detect that the data indicate an incompatibility with the null hypothesis when it is not true is known as the “power” of the test procedure (in our case, the ability to find a trend when one does exist). The probability of a Type II error equals (1 – power). It is important to note that the power of a test depends on “how false” the null hypothesis is; for example, a test could have relatively low power and still identify a strong trend, but would need to have relatively high power to identify a slight trend. The power of a test is often a consideration made during the design phase of a scientific study, especially when choosing the sample size. However, the data in TOAR is limited to the time periods for which the monitors were operating, and thus power was not a major consideration.

The choice of an alpha of 0.05 as a significance level is arbitrary. TOAR selected this specific level because of the very large number of sites and metrics which were to be tested. Hence, a common point of reference was needed for summarization and comparison purposes, and the 0.05 level is quite common in the literature. However, TOAR is not wedded to a ‘Yes/No’ outcome based on a 0.05 level. TOAR retains the actual calculated *p*-values in its database. Thus, any researcher who prefers to use either a different level or, indeed, wishes to work with the individual *p* -values for an analysis has the opportunity.

For the TOAR assessment, the following terminology is used when describing trend results:

- a trend result associated with a *p*-value of 0 to 0.05 is a statistically significant trend;
- a trend result associated with a *p*-value of 0.05 to 0.10 is referred to as indicative of a trend;
- a trend result associated with a *p*-value of 0.10 to 0.34 is described as having a weak indication of change; and
- a trend result associated with a *p*-value of 0.34 to 1 is described as weak or no change.

The final two categories listed above are shown for informational purposes only, and researchers are strongly cautioned against associating *p*-values greater than 0.10 with statistical significance.

An important consideration in selecting tests for assessing trends in the TOAR program was the requirement that the same statistical methods be applied across the thousands of measurement sites in the TOAR database and the various metrics to be analyzed. The large amount of data to be characterized precluded a detailed review of the data from every site/metric combination to determine (1) an appropriate analytical functional form that fit the data and (2) whether a regression approach (either linear or nonlinear) would be appropriate. The nonparametric Mann-Kendall (M-K) test (to test for significant trends) and the Theil-Sen (T-S) estimator (for estimating the magnitude of the trend) were selected. The T-S and M-K methods require (1) no assumptions regarding functional form or statistical distribution for the data and (2) are resistant to outliers, and (3) do not require consideration of whether trends are linear or nonlinear.

For focused analyses involving subsets of sites, TOAR recognized that parametric statistical tests, such as linear regression, can be applied if the required assumptions were met. For example, standard linear regression necessitates: (1) a linear model is appropriate to describe the data and (2) the variable is normally distributed, (3) has constant variance, and (4) the data are independent observations. The assumption of constant variance may be inappropriate, especially for ozone data because the inter-annual variability tends to decrease as precursor emissions are reduced and levels approach background levels. Ozone time series, based on less than annual data values, can have significant autocorrelation and ozone metrics are often not normally distributed. Ignoring the above assumptions can lead to an incorrect conclusion about the statistical significance and the associated confidence intervals of the regression parameters, thus resulting in significant uncertainty regarding the conclusions for the trends analyses.

In this section, we discuss some of the statistical approaches available for characterizing trends. Both nonparametric and parametric approaches for assessing trends are discussed, including the advantages of the various approaches. Examples are provided that describe how violations of key assumptions affect the estimates of the significance of the trends, as well as the magnitude of change. Section 3.3 discusses data completeness criteria.

### 3.1. Statistical approaches for characterizing trends

Various statistical tests have been used to identify statistically significant trends and the rate of change associated with these trends (e.g., Oltmans et al., 2006; Sicard et al., 2009, 2013; Lefohn et al., 2010a; Cooper et al., 2012; Wilson et al., 2012; Derwent et al., 2013; Munir et al., 2013; Oltmans et al., 2013; Parrish et al., 2013; Parrish et al., 2014; Simon et al., 2015; Malley et al., 2015). Because TOAR is using the nonparametric M-K and T-S methods as the preferred approach for characterizing trends, they will be discussed first.

#### 3.1.1. Nonparametric statistical tests

##### 3.1.1.1. Testing for significance of a trend

The Mann-Kendall (M-K) nonparametric test (Mann, 1945) is utilized to test for a significant trend. Advantages of the M-K test are:

- No distributional assumption is made;
- No assumption of any specific functional form for the behavior of the data through time is made. Thus, the M-K test is universally applicable across all sites, seasons, and different continuous summary TOAR exposure metrics (e.g., percentiles, means, and cumulative indices, such as the SOMO10, SOMO35, W126, and AOT40 exposure metrics); and
- The M-K test is resistant to the effects of outlying observations. The results are not unduly affected by particularly high or low values that occur during the time series.

Outliers are fairly common in air quality and other environmental data. Because the M-K nonparametric test targets the median instead of the mean, it is more robust to outliers than parametric tests. The M-K test requires fewer *a priori* assumptions about the data than the application of other statistical techniques. As indicated above, one advantage of using the M-K test is its universal applicability.

However, the M-K test, similar to other approaches, can be problematic when using count metrics, such as the number of days during the year equal to or above a specific value. Extensive ties in counts may cause problems. Tables exist for the M-K test in its exact form and an asymptotic version is also available (Hollander et al., 2013). The M-K test explicitly accounts for ties both in the test statistic itself and its variance (and hence the *p*-value). In the presence of ties, the test statistic is calculated to explicitly account for these. The exact version may be applied directly. The asymptotic version requires an adjustment of the variance of the test statistic to account for the tied values.

Another approach to analyzing count data might be, depending upon the specific question(s) under investigation, to convert each count into a value representing a fraction. A mathematical transformation might then be applied to the converted data points and an appropriate statistical approach used on the transformed data. Other options could include the use of logistic regression or Poisson regression.

The same characteristics noted above for the M-K test apply to a very similar nonparametric approach, the Daniels test, which can be implemented using the Spearman correlation coefficient (Daniels, 1950; Gibbons and Chakraborti, 2011). Direction of the trend is indicated by the sign of the correlation coefficient, and statistical significance is indicated by whether the correlation coefficient is different from zero. Simon et al. (2015) used this procedure to assess ozone trends in the 5^th^, 25^th^, 50^th^, 75^th^, and 95^th^ percentiles of mean daily maximum 8-h average ozone concentrations at US monitoring sites.

While these two nonparametric methods are similar, TOAR has selected the M-K test because the underlying statistic, Kendall’s correlation coefficient, has some slight advantages over the Spearman correlation coefficient in terms of interpretability, sensitivity to the distribution of the variable being analyzed, simplicity of the background theory, and faster asymptotic convergence (Kruskal, 1958; Kendall and Gibbons, 1990; Gibbons and Chakraborti, 2011).

##### 3.1.1.2. Estimating the magnitude of a trend

For estimating the magnitude of a trend, the Theil-Sen (also called Sen-Theil, Theil, or Sen) estimator is used (Theil 1950a, 1950b, 1950c; Sen, 1968) by TOAR. It possesses the same attributes described above for the M-K test (i.e., there are no distributional or functional form assumptions and the estimator is resistant to outliers). The Theil-Sen (T-S) estimator, similar to the M-K technique, is also universally applicable. In cases where simple linear regression is appropriate (i.e., assumptions are met), the slope of the regression line and the T-S estimator are asymptotically equivalent.

TOAR’s approach of first testing for the existence of a trend with the M-K test and then estimating the magnitude of the trend with the T-S statistic will generally perform well. However, as suggested above, count data may yield somewhat problematic results for some data sets. For example, if one has counts which are all zero for the vast majority of the beginning or end of the time series and a few monotonically changing counts at the end or beginning, respectively, then the M-K test may yield a statistically significant trend, but the T-S estimator may be zero. It is possible to interpret such a counterintuitive result of a statistically significant trend that is estimated to be zero as indicating evidence of a trend that is very small. On the other hand, one can examine such a case further by using a different or modified estimator of the trend size (Lefohn et al., 2017).

##### 3.1.1.3. Trends in data with seasonality

For examining trends in a time series that contains seasonality (e.g., winter, spring, summer, and fall; warm season/cold season), the seasonal Kendall test and its associated modified T-S estimator can be applied (Hirsch et al., 1982). Both approaches are modifications of the M-K test and the T-S estimator described earlier. To test for trend in individual seasons, the M-K and T-S methods can be used to yield results for each season. To account for seasonality, while testing for overall trend through the entire time period, the seasonal Kendall test can be used. For each season, one calculates the desired metric. Using the seasonal metric, comparisons are made within each season across the time period of interest and then the results are appropriately combined to provide one trend test and magnitude estimate over the entire time period. An advantage of using the seasonal Kendall test when seasonality exists is to improve one’s ability to detect an overall trend through the entire time period.

#### 3.1.2. Parametric statistical tests

It is worth clarifying the distinction between a parametric linear regression approach and the underlying functional form of the data. For example, one might propose the following nonlinear functional form: Y = b_0_ + b_1_ x + b_2_ × ^2^. This is a nonlinear function in terms of the predictor variable x, but it is linear in the parameters b_0_, b_1_, and b_2_, which are to be estimated. Linear regression could be used to estimate the parameters for this function. The model Y = b_1_ x/(b_2_ + x) is a case of a model which is nonlinear in both the predictor variable and the parameters.

The parametric linear regression approach for assessing the behavior of the data is familiar, widely known, straightforward to apply, and often used by researchers. The approach is readily available in almost all statistical packages. If the underlying functional form of the model is correct, the overall F test indicates whether some of the (non-intercept) parameters are zero, that is, whether the model has much explanatory power or not given the variability in the data; the coefficient of determination or R^2^ measures how much explanatory power the model has. The Student’s *t* test may be used to evaluate the significance of each individual parameter. However, while the F test and R^2^ can provide some guidance as to the appropriateness of the underlying model, neither a significant F test nor a large R^2^ can be taken as verification that the underlying model is correct. If the underlying functional form is a straight line, the trend is given by the slope of the regression line. If the underlying functional form is not a straight line, the interpretation of the result using this trend test may be unclear.

The use of linear regression to assess trends in observed values potentially can be problematic. As indicated above, it is important when applying the technique that the underlying assumptions for linear regression analyses are met. The specific assumptions of most concern are (1) the underlying functional form is appropriate to describe the data and (2) the errors are normally distributed, (3) the errors have constant variance, and (4) the data are independent observations. It is important that diagnostics (e.g., residual analysis, cross validation) be performed to confirm the validity of the method’s assumptions. When the number of observations is small, the assumption of normally distributed errors with constant variance becomes difficult to confirm. If the underlying data are not normally distributed about the regression line or are not independent, the statistical conclusions reached (i.e., either significant or not) are questionable.

Other parametric approaches also exist. For example, nonlinear regression of some form may be considered. However, the nonlinear regression approach generally entails the same difficulties as noted for the linear regression approach. In addition, nonlinear regression approaches are typically more complex and difficult to implement.

If the assumptions are met, a parametric approach will generally be more powerful than the nonparametric approach. However, similar to the M-K test, the linear regression test can be problematic when working with count metrics (e.g., the number of days during the year equal to or above a specific value).

#### 3.1.3. Additional approaches

As noted in the introduction to this section, TOAR seeks to investigate trends at a very large number of sites across broad geographic areas and for a wide variety of exposure metrics. For these reasons, TOAR is utilizing the M-K and T-S nonparametric techniques. However, if one desires to examine trends in more depth at specific sites, different statistical methods may afford more power and/or allow more detailed analysis. For example, in cases when the data are either Gaussian or can be transformed to be nearly Gaussian (e. g., by taking the logarithm of the data), statistical approaches described by Box et al. (2015) may be used to derive trends in the presence of autocorrelation and estimate confidence intervals about those trends (Box et al., 2015; Weatherhead et al., 1998). Note, however, that the length of the annual time series considered by TOAR (e.g., 2000–2014) may make the reliable estimate of autocorrelation problematic (Box et al., 2015). Regardless of whatever methodology is chosen for a more detailed site-level analysis, it is important that the assumptions required by the technique(s) be met.

As indicated above, autocorrelation is a potential concern when conducting trends analyses. While autocorrelation is not generally anticipated to be a concern for trends based on annual metrics, an assessment was undertaken to identify the degree of autocorrelation that may be present in the trends analysis presented in TOAR. Trends computed using the T-S technique described above for 14 TOAR ozone exposure metrics at 196 US and 276 EU sites over the period 2000–2014 for the US and 2000–2013 for the EU were tested for the presence of lag-1 autocorrelation (Kendall’s Tau statistic) using data obtained from the case study (Lefohn et al., 2017) described in Section 4. At the 5% significance level, only 4% of the EU sites and 2% of the US sites exhibited autocorrelation statistically significantly different from 0. In addition, only 2% of the EU sites and 0.5% of the US sites had autocorrelation greater than 0.5, and no sites in either region had autocorrelation greater than 0.7. The level of autocorrelation present was fairly consistent across the 14 metrics. Based on this analysis, evidence of worrisome levels of autocorrelation for the annual metrics was not observed over the 15-year period used in TOAR.

For estimating ozone trends for the various metrics in their analyses, Munir et al. (2013) used a variety of methods including: quantile regression, T-S technique, changepoint analysis, and a generalized additive models approach that combined a smooth function of time with loess. Munir et al. (2013) illustrate the large number of approaches one may use for characterizing trends. However, several of these techniques are highly dependent on the specific data with which one is dealing. This dictates that the trend analysis must then be “fine-tuned,” potentially on a case-by-case basis. So while one may employ the same general approach for the analysis across all sites, the approach may have to be implemented differently from site to site. Thus, one loses the universal applicability of the M-K and T-S methods adopted for the TOAR analyses.

### 3.2. Examples of results that compare nonparametric and parametric statistical tests

Both the parametric and nonparametric approaches assume independent observations and can tolerate missing data within reason. For the purposes of TOAR, when comparing trends on a site-by-site basis, based on its universal applicability, the nonparametric approach is utilized. Assumptions required for using a parametric approach may not be met when assessing trends at each monitoring site, with the result that some sites would have to be rejected. This would result in compromising a comparison of trend patterns across sites. The nonparametric approach would likely apply at any site.

**Figure 4:**
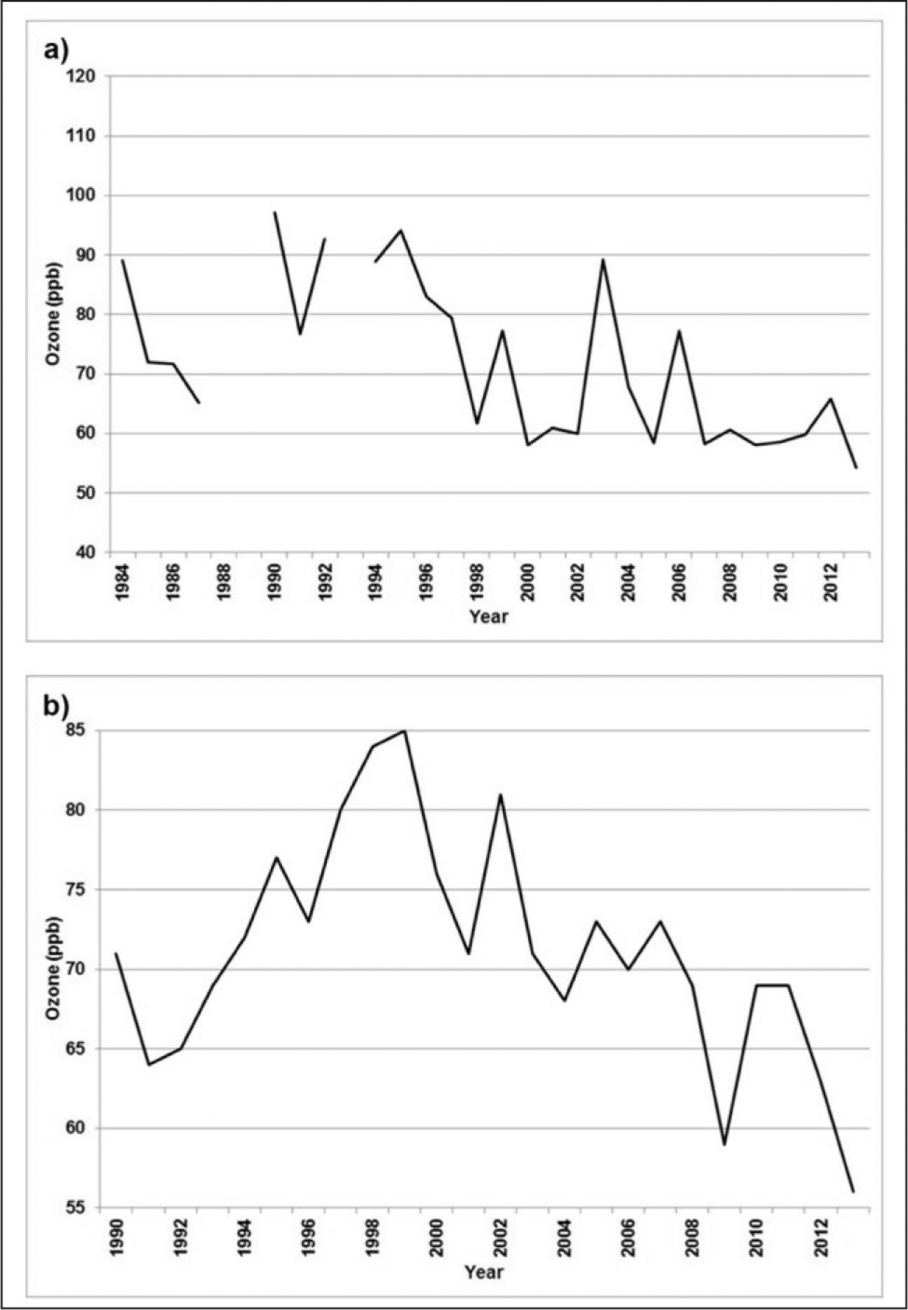
Time series for **(a)** Harwell, UK (1984–2013) for the 4^th^ highest MDA8 level and **(b)** Look Rock, Tennessee (1990–2013) for the 95^th^ percentile. DOI: https://doi.org/10.1525/elementa.279.f4

We have selected data from a site at Harwell, UK, and a site at Look Rock, Tennessee for comparing nonparametric and parametric statistical test results. For these illustrations, the metrics used are the annual fourth highest daily maximum 8-h average level for Harwell and the annual 95^th^ percentile for Look Rock. The time series for both sites are displayed in Figure 4a and **4b**.

For the Harwell site, data from 1984–2013 were tested for trend using: (1) the M-K test, and (2) by regressing a straight line through the data with the year as the predictor variable. For the nonparametric approach, the magnitude of the trend was estimated by the T-S estimator and for the parametric test by the slope of the regression line. In addition, 95% confidence intervals for the trend magnitudes were calculated. Over this 30-year time period, there were 27 years of valid data.

At Harwell, both methods yielded similar results. A statistically significant (p < 0.01) trend was found using each technique. Using the linear regression approach, the basic assumptions for the method did not appear to be seriously violated, although there was some evidence of higher variability for the larger values of the fourth highest daily maximum 8-h value. The lack of constant variance may have affected the significance level to some extent and may have contributed to the low R^2^. The results are summarized in Table 2 below.

Using the 95^th^ percentile level, a similar statistical comparison approach was employed for a site located at Look Rock, Tennessee for the period 1990–2013 (24 valid years of data). Table 3 summarizes the outcome.

On their face, the nonparametric and the parametric methods yielded similar results for the Look Rock site. Neither method reported a trend significant at the 5% level (Table 3). However, the linear regression approach suffered from model mis-specification. That is, a straight line was clearly not the appropriate underlying functional form to use (see Figure 4b). The low R^2^ value reflected this. Therefore, the results associated with applying the regression approach were not reliable.

### 3.3. Data capture

Data capture (i.e., the amount of valid hourly data available in a given sampling period in which aggregation is applied) may have strong impacts on the derived metrics or trend estimate. It is easily seen that a measurement series consisting only of a few nighttime measurements during winter will not reflect photochemical ozone maxima which occur during daytime in summer. Consequently, any evaluation of metrics, which focus on the higher part of the distribution from such data series, would be meaningless. In reality, the vast majority of ozone measurement series are more or less complete so that the derived metrics and trend parameters can generally be assumed to be robust. However, there is a margin of uncertainty in this statement of “more or less”, and the reader should be aware of the possible implications of incomplete data series.

**Figure 5:**
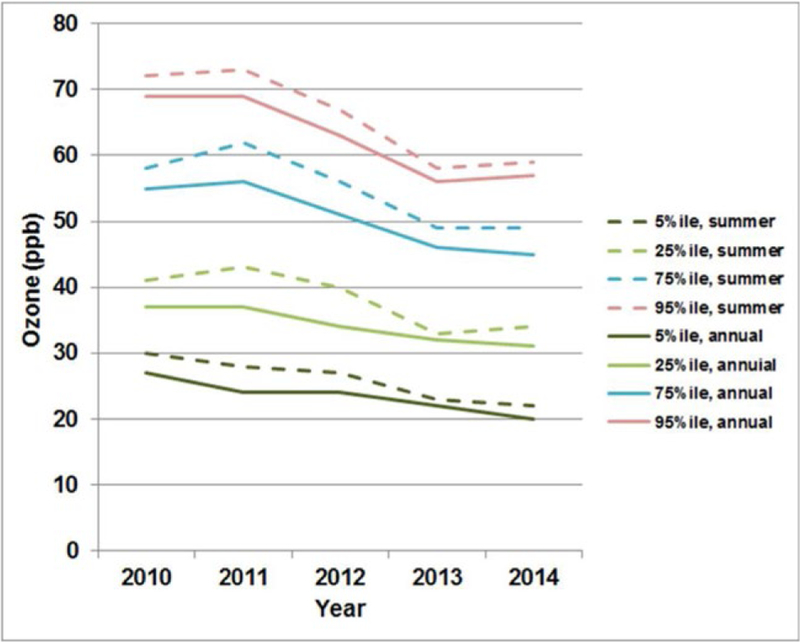
Comparison of selected annual percentiles of ozone levels at Look Rock, TN during the summer months (April–September) with the same percentiles derived from the entire annual data. DOI: https://doi.org/10.1525/elementa.279.f5

Typical examples of incomplete data are associated with many US regulatory monitoring stations, which are required to operate only during the so-called “ozone season” (i.e., a varying period of several months during the summer depending on state, but also varying over the years). Given the general tendency of ozone levels to be higher in summer than in winter, the evaluation of annual statistics at such sites would provide different results. This can be demonstrated by comparing for example selected percentile values evaluated during the summer months with those evaluated over the full year from a station with full annual coverage (Figure 5).

Data capture also matters if we want to assess the robustness of the extreme values in a data set. Our confidence in a reported 4^th^ highest daily 8-hour maximum in a given year would obviously be greater if this metric were evaluated from a data series that has valid measurements every day rather than only every second or third day. The statistically interesting questions “How different (i.e., incorrect) is the magnitude of a given metric if x% of data are missing?”, or “What is the probability that the magnitude of a given metric is incorrect by a certain amount if x% of data are missing?” have not yet been addressed systematically for ozone observations, or, more generally, for environmental data sets.

Based on established practices and some tests on selected data series, a general data capture criterion of 75% was applied in all TOAR analyses. This data capture threshold is applied on various levels. For example, to calculate a valid 4^th^ highest daily 8-hour maximum value in a year, there must be 75% of hourly values available in each (running) 8-hour averaging interval, then there must be 75% of valid 8-hour intervals during a day, and finally, 75% of valid days in a year. The data capture criteria for each of the TOAR exposure metrics are summarized in the supplement of *TOAR-Surface Ozone Database*.

If the data capture at a site is poor, the data should not be combined with another site’s data unless there are circumstances that can be well documented. For example, some time series consist of two or more partial datasets that are stored individually in separate networks. In these cases, the data series are combined. However, in other situations, such as when stations with long records are relocated, extensive statistical analyses should be undertaken to confirm that the merging of the datasets is appropriate. TOAR has made the decision to generally not combine data from different sites but a few exceptions were made and are noted in the TOAR database.

Performing trend analyses by season versus an entire year of data is an important consideration. Some states in the US are required to only monitor during the “ozone season”, which historically have been as short as June-September and as long as January–December. TOAR requires 75% of the full calendar year data for all of the annual TOAR metrics. For sites in the TOAR database that do not operate year-round, missing values are reported for annual exposure metrics. For TOAR metrics determined for a summer period, the 6-month April–September (Northern Hemisphere) and October–March (Southern Hemisphere) periods are used. The TOAR database produces daily and monthly exposure metrics and thus, users who wish to create their own season definitions have the ability to do so. A complete description of the data validation criteria is described in *TOAR-Surface Ozone Database*.

## 4. Response of exposure metrics to changes in ozone distributions

Hourly ozone levels are used to calculate the magnitude, spatial distribution, and trend for various exposure metrics associated with human health, vegetation, and climate change. Exposure metric trends are associated with changes in the frequency of hourly average levels across an ozone distribution. As indicated in Section 1, different metrics used for assessing human health or vegetation risks can have different long-term trends (i.e., different metrics can increase, show no change, or decrease) under identical changes in the ozone concentration distribution over time (Karlsson et al., 2007, 2017; EEA, 2009; Tripathi et al., 2012; Li et al., 2014; Paoletti et al., 2014; Malley et al, 2015; Lefohn et al., 2017). Besides estimating risk to human health and vegetation, this has significant relevance for assessment of how changes in emission controls have resulted in changes to ozone impacts on human health and vegetation.

**Figure 6:**
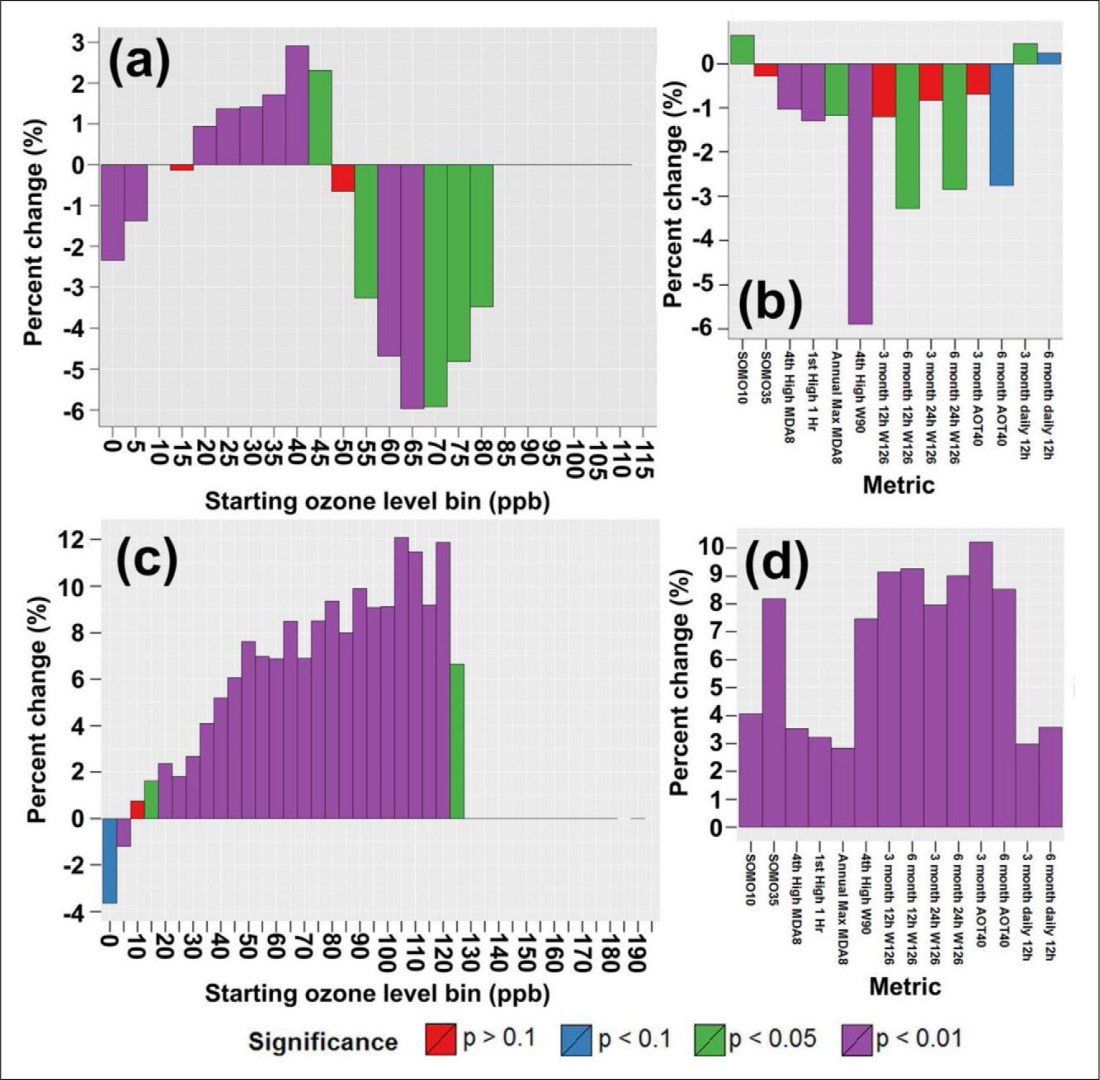
Theil-Sen (%/year) trend for **a)** hourly ozone levels in each bin, and **b)** 6 human health and 8 vegetation ozone metrics for a site at Glazebury, UK between 1989 and 2013, and **c)** hourly ozone levels in each bin, and **d)** 6 human health and 8 vegetation ozone metrics for a site at Yuen Long, Hong Kong, China between 1995 and 2015 (significance determined by the Mann-Kendall test at p < 0.05). (Data characterized as per Lefohn et al., 2017). DOI: https://doi.org/10.1525/elementa.279.f6

As changes in emissions occur, concentrations within a specific part of a distribution can change at a different rate and/or direction than other parts of the distribution. This could result in different metrics providing different responses to emissions controls. An illustrative example for Glazebury, a rural site in the UK, shows that for a common trend across the hourly ozone concentration distribution (Figure 6a), both increasing and decreasing statistically significant trends in some exposure metrics at p < 0.05, and no significant trends in others are observed (Figure 6b). In this section, we discuss how several of the exposure metrics applied in TOAR papers (this issue) behave in response to changing ozone distributions over time. Assessment of temporal changes in the ozone distribution and related changes in the metrics described in this section use the Mann Kendall (M-K) and Theil-Sen (T-S) statistics as discussed in Section 3.

To further demonstrate the relationship between distribution changes and human health and vegetation metrics, in Section 4.1 we summarize the results from a case study (Lefohn et al., 2017). The study focused on assessing how changes in the ozone distribution profile in regions where emissions of ozone precursors have decreased (i.e., US and EU) and increased (China) influenced temporal trends in a set of human health and vegetation exposure metrics similar to those selected for use by TOAR at monitoring sites in these regions. For this purpose, trends in 14 human health and vegetation TOAR exposure metrics were examined at 276 EU, 196 US, 3 Mainland China, and 6 Hong Kong, China sites. We then extend the analysis of Lefohn et al. (2017) by comparing the trend patterns of other TOAR metrics between 1995 and 2014 with patterns observed in the case study for gaining insight about the relationships of TOAR metrics among one another (Section 4.2).

### 4.1. Summary of ozone exposure metrics trend case study

**Figure 7:**
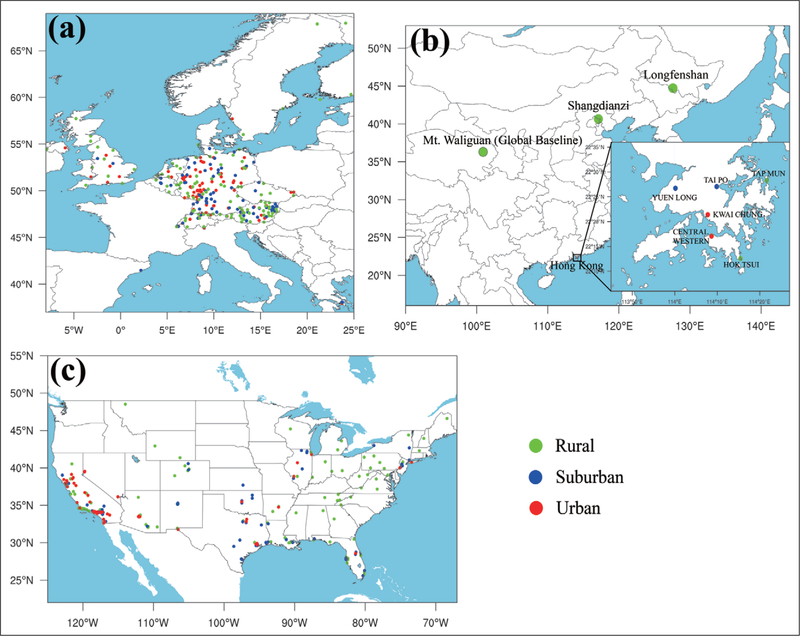
Map of **(a)** EU, **(b)** mainland China and Hong Kong, China, and **(c)** US sites selected for the study (Lefohn et al., 2017). DOI: https://doi.org/10.1525/elementa.279.f7

Figure 7 identifies the locations of the sites used in the case study. The subset of 14 metrics (Table 4) reflect the variation of some of the TOAR metrics in terms of their focus on relatively high, moderate, and low ozone levels.

#### 4.1.1. Identifying distinct hourly ozone distribution trend types

The case study identified changes in hourly average ozone distributions into ten distinct trend type patterns (Lefohn et al., 2017). These patterns were:

- **Trend Type 0:** No trend.
- **Trend Type 1:** Both ends of the distribution shift toward the center. (Decreasing frequency of high and low levels).
- **Trend Type 2:** Low end shifts upward but high end does not change. (Decreasing frequency of low levels; increasing frequency of middle levels).
- **Trend Type 3:** High end shifts downwards but no change at lower end (Decreasing frequency of high levels; increasing frequency of middle levels).
- **Trend Type 4:** Entire distribution shifts downwards (Decreasing frequency of high levels, increasing frequency of low levels).
- **Trend Type 5:** The distribution shifts from the center toward both the high and the low ends of the distribution. (Increasing frequency of high and low levels).
- **Trend Type 6:** The middle of the distribution shifts downward but the high end does not change. (Increasing frequency of low levels, decreasing frequency of middle levels).
- **Trend Type 7:** The middle of the distribution shifts upward but the low end does not change. (Increasing frequency of high levels, decreasing frequency of middle levels).
- **Trend Type 8:** Entire distribution shifts upwards. (Increasing frequency of high levels, decreasing frequency of low levels).
- **Trend Type X:** Complex trends that do not fall into any of the categories listed above. It is not possible to categorize portions of the ozone distribution into “low”, “middle”, and “high” for this trend type because the directions of the trends shift more than two times across the distribution.

For assessing the trend behavior of median concentrations, Trend Type 1 sites (i.e., compression from both ends toward the center) were grouped in the study into three subcategories: (1) “1a” sites had increasing median concentrations; (2) “1b” sites had no trend in the median; and (3) “1c” sites had decreasing median concentrations.

Various shifts in ozone distributions occur as differing emissions changes occur. For example, Figure 6a illustrates the shifts that occurred between 1989 and 2013 as a result of emissions reductions in the hourly average ozone levels for the rural Glazebury site. Both ends of the distribution shift toward the center with decreasing frequencies occurring at the high end (55 to 85 ppb) and low end (0 to 10 ppb) of the distribution with increasing frequency of hourly ozone levels between 20 ppb and 45 ppb. This Trend Type 1 site is further designated as Trend Type 1a because the median concentration increased (data not shown for median increase). In contrast, Figure 6c illustrates the entire distribution shifting upwards (Trend Type 8) as a result of regional emission increases for a suburban site at Yuen Long, Hong Kong, China between 1995 and 2015.

**Figure 8:**
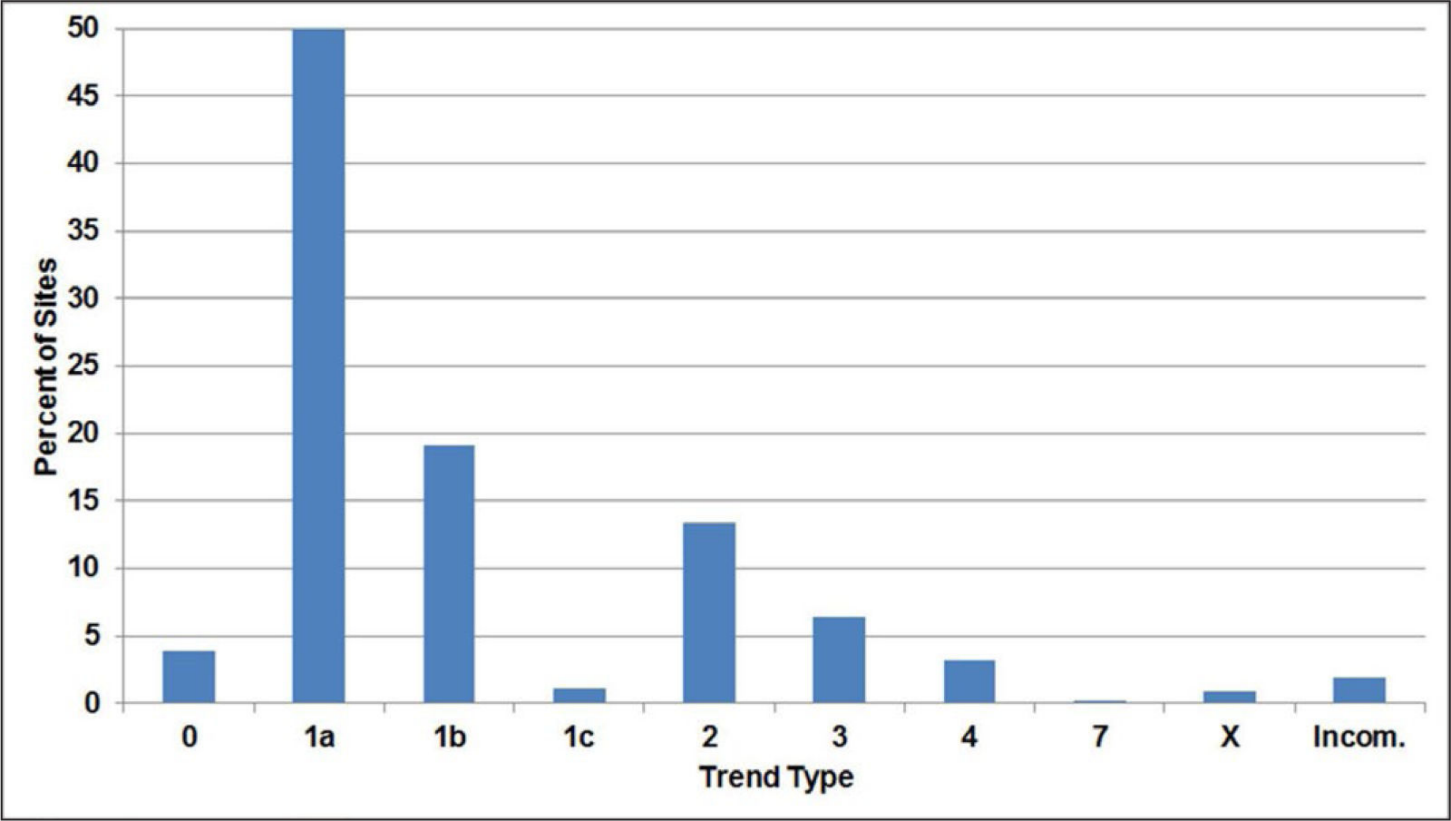
Percent of combined EU and US sites that exhibited specific trend types that occurred. (Data results summarized from Lefohn et al., 2017). DOI: https://doi.org/10.1525/elementa.279.f8

The predominant pattern for the 276 EU and 196 US sites characterized in the case study was the shifting of high and low levels toward the center (Trend Type 1) (Figure 8). Seventy percent of the combined EU and US sites experienced this pattern and 71% of those sites had increasing median ozone levels (i.e., Trend Type 1a) (Figure 8). A higher proportion of US sites were classified as Trend Type 1 (81% of all US sites compared with 60% of all EU sites), and as the Trend Type 1a sub-group (61% of all US sites, 43% of all EU sites). As described in Section 1, this trend type is consistent with behavior expected in regions, such as the EU and US, which have implemented large decreases in regional NO_x_ emissions. Because of precursor emission increases in mainland China, some sites in mainland China and Hong Kong exhibited the middle of the distribution shifting upwards but the low end not changing; for other sites in China, the entire distribution shifted upwards. All nine of the Chinese sites characterized in the case study were either Trend Types 7 (increasing frequency of high levels), Trend Type 8 (increasing frequency of high ozone, decreasing frequency of low ozone), or Trend Type X (complex trends that could not be categorized (Lefohn et al., 2017). The characteristics for the 9 Chinese sites are described in Lefohn et al. (2017).

#### 4.1.2. Response of exposure metrics to changes in distribution patterns

Table 4 identifies the TOAR metrics selected in the case study for characterizing the relationship between exposure metrics and changes in distribution patterns. The metrics listed in Table 4 are a subset of the human health and vegetation effects metrics described in Table 1. Details about the months associated with the averaging or accumulation periods in the seasonal exposure metrics (i.e., 3- and 6-month periods) are found in *TOAR-Vegetation* and http://www.igacproject.org/activities/TOAR. The 3-month season is defined to be representative of the wheat growing season in different climate zones as specified in *TOAR-Vegetation* and http://www.igacproject.org/ activities/TOAR.

**Figure 9:**
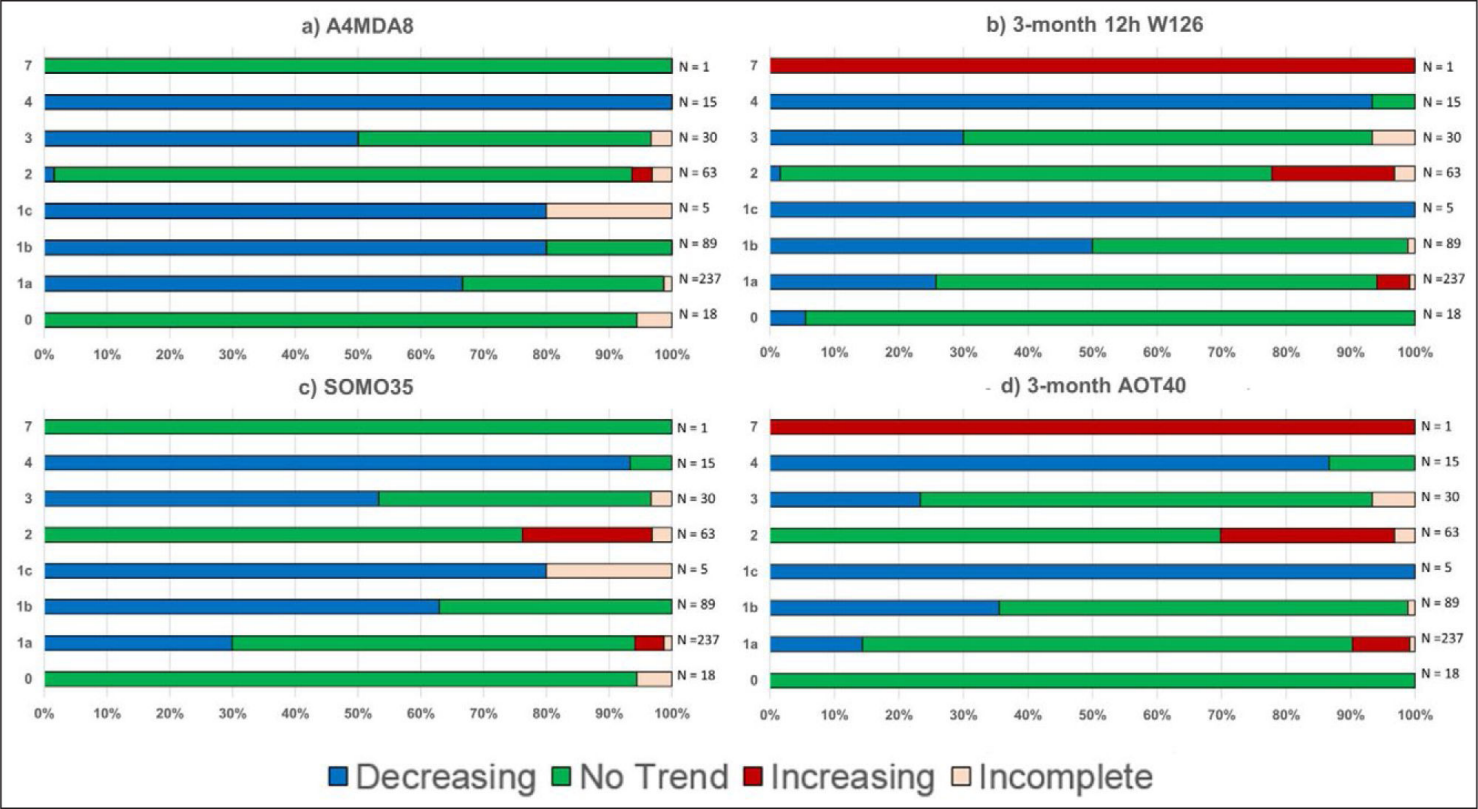
Percentage of EU and US sites combined in each trend type (e.g., 0, 1a, 1b, etc.) with trends in **(a)** A4MDA8, **(b)** 3-month 12-h W126, **(c)** SOMO35, and **(d)** 3-month AOT40. Summarized results from Lefohn et al. (2017). Trend Types 5, 6, and 8 did not occur and Trend Type X occurred infrequently. DOI: https://doi.org/10.1525/elementa.279.f9

**Figure 10:**
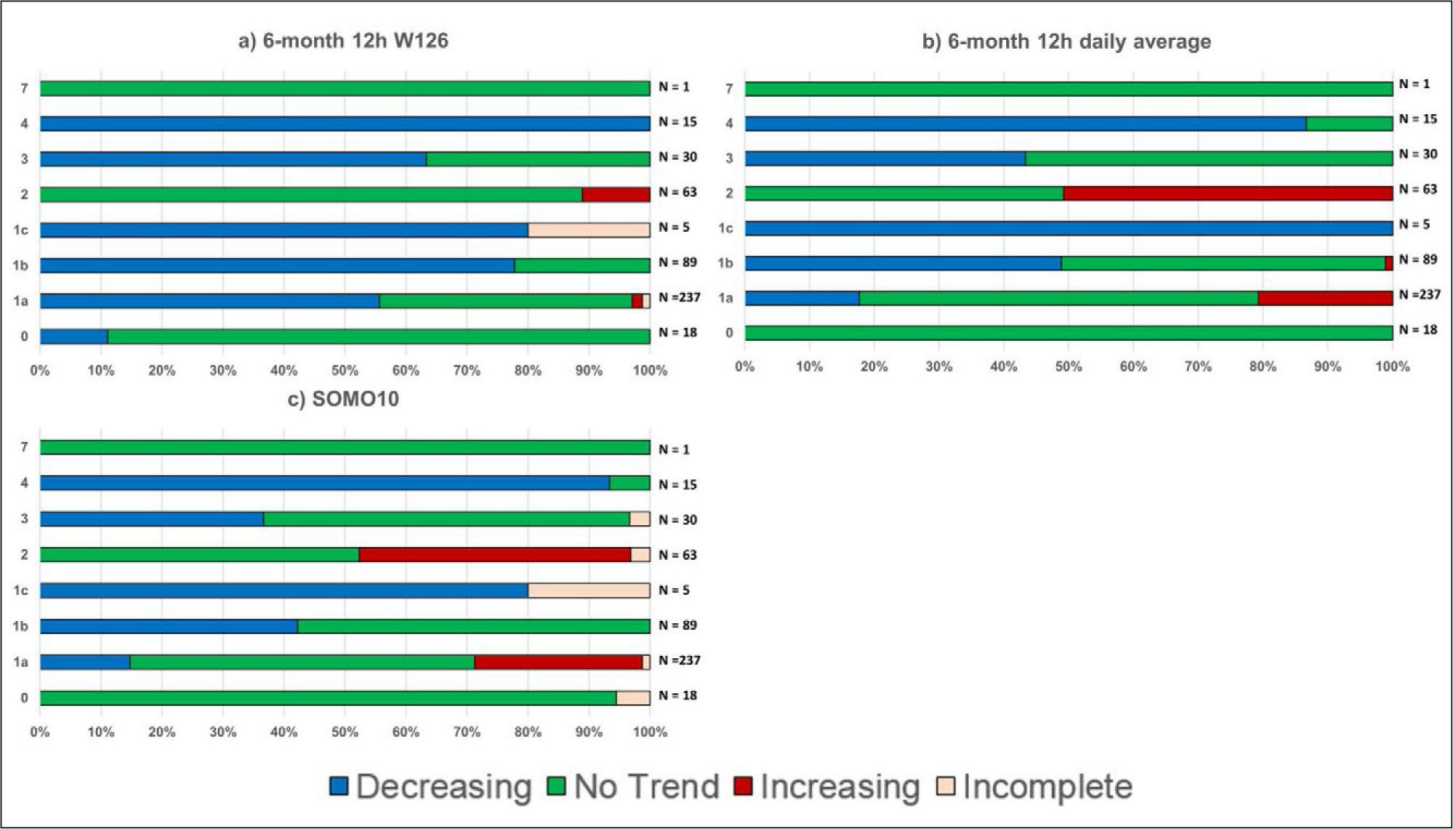
Percent of EU and US sites combined in each trend type (e.g., 0, 1a, 1b, etc.) with trends in **(a)** 6-month 12-h W126, **(b)** 6-month 12-h daily average, and **(c)** SOMO10. Summarized results from Lefohn et al. (2017). Trend Types 5, 6, and 8 did not occur and Trend Type X infrequently. DOI: https://doi.org/10.1525/elementa.279.f10

The shifting concentrations within the distribution at EU and US sites resulted in varying trend patterns for the exposure metrics (i.e., some decreased, while others increased under the same change in ozone concentration distribution) (Figures 9 and 10). These patterns varied across sites. Analysis of the EU and US sites showed that for metrics determined solely by the highest ozone levels (e.g., A4MDA8), decreasing trends were calculated at the majority of sites at which the frequency of high levels decreased, regardless of changes occurring across other parts of the ozone concentration distribution. For example, at 70% of all sites assigned as Trend Type 1a, 1b, 3, or 4, A4MDA8 decreased (Figure 9a). The A4MDA8 showed no trend at the vast majority of the uncommon Trend Type 2 sites (i.e., low end shifts upward but high end does not change) (Figure 9a).

Trends in metrics influenced by both moderate and high hourly levels were consistent with the trend types (Figures 9b, c, d and 10a). For example, the SOMO35 and 6-month W126 metrics decreased at a majority of Trend Types 3 and 4 sites (decreasing frequency of high hourly ozone levels), with no trend calculated at the other sites. Conversely, at Trend Type 2 sites (decreasing frequency of low hourly ozone levels), these metrics either increased or showed no trend. At Trend Type 1 sites, the opposing changes (i.e., high levels shifting downward and low levels shifting upward) resulted in a greater variety in the trends in these metrics. A small fraction of Trend Type 1a sites had increasing SOMO35 and 6-month W126 values, while a much larger number of these sites experienced decreases in those metrics. The fraction of decreasing trends within Trend Type 1 sites was larger for subcategories that had no trend in the median (1b) or decreasing median values (1c) than for Trend Type 1a sites which experienced increasing median values.

Finally, trends in the metrics influenced primarily by moderate levels (i.e., 3- and 6-month daily 12-h averages), and the metric determined by low, moderate and high levels (i.e., SOMO10), were similar to trends in the metrics that focused on moderate and high levels except that the relative proportion of decreasing, increasing, and no trends differed at Trend Type 1 sites (e.g., compare SOMO10 to SOMO35 in Figures 9 and 10).

For the sites in mainland China and Hong Kong, all the exposure metrics increased or showed no trend in the case study as a result of the upward shifts in either relatively high ozone levels or across the entire distribution (e.g., please see Figure 6c and 6d).

Identifying the influence of emissions changes on trends in various exposure metrics is not necessarily straightforward. The predominant pattern in the case study **for** the EU and US sites, where substantial reductions in ozone precursor emissions have occurred (EEA, 2015; US EPA, 2014b), was the shifting of high and low levels toward the center (Trend Type 1) with median concentrations increasing (Trend Type 1a) (Lefohn et al., 2017). Similarly, median concentrations increased at Chinese sites where emissions of NO_x_ have increased until recently in mainland China (Duncan et al., 2016), while in Hong Kong, there have been large reductions in local emissions of both NO_x_ and VOC since 1997 (http://www.epd.gov.hk/epd/ english/environmentinhk/air/data/emission_inve. html#emission_trends). This highlights the fact that ozone levels are the result of complex chemical and physical atmospheric processes and are impacted by spatially and temporally heterogeneous local, regional, and large-scale emissions changes.

### 4.2. Comparison between trends patterns described in the case study and trend patterns in the metrics in the TOAR database

In the *TOAR-Health* and *TOAR-Vegetation*, the metrics described in Table 4, as well as other metrics (see Section 2), are used to describe spatial variation and long-term trends over a fixed time period (e.g., 1995–2014 and 2000–2014) across sites globally. Using data from the TOAR database with appropriate data capture as specified by *TOAR-Surface Ozone Database*), we investigate whether trend patterns of human health and vegetation metrics associated with changes between 1995 and 2014 for all relevant TOAR sites were consistent with the trend patterns (at the p < 0.05 level) observed in the case study described in Section 4.1. Additionally, mean and median concentrations determined on an annual and summer seasonal (April–September NH; October–March SH) basis are included in the comparison. For each site, the trend direction for each metric was identified and compared among all the other exposure metrics. For each pair of metrics, Tables 5 and S-4 (in Supplemental Material) summarize the proportion of TOAR sites for which the trends in both metrics are in the same direction (i.e., both decreasing, both increasing, or both with no significant change at the p < 0.05 level) between 1995 and 2014. This provided quantitative information on which metrics behaved similarly and which metrics did not.

Those exposure metrics influenced by similar level ranges tended to have consistent trend patterns (Tables 5 and S-4). For example, the 4^th^ highest daily maximum 8-h and 4^th^ highest W90 exposure metrics, which are influenced by the highest hourly levels, exhibited trends in the same direction between 1995 and 2014 at 92% of sites analyzed in the TOAR database; neither of the two metrics exhibited a strong relationship with the annual/seasonal median or mean concentrations. On the other hand, the trends for the 4^th^ highest daily maximum 8-h metric and the SOMO10 metric were only in the same direction at 55% of sites. While the 4^th^ highest daily maximum 8-h metric is influenced by changes in the higher levels, the SOMO10 metric is influenced by changes occurring across low, moderate, and high levels. In contrast, between 64% and 83% of sites had trends in the same direction for SOMO10 and the various mean and median concentrations. Based on the results summarized in Tables 5 and S-4, all metrics included in the analysis were then grouped based on the similarity of trend patterns across the TOAR database sites (proportion of sites in agreement ≥ 80%). The groups of metrics with similar trends patterns to each other, and dissimilar patterns to metrics in other groups are as follows:

Human health metrics fell into two distinct groups:

1. The 4^th^ highest daily maximum 8-h during the year (4^th^ dma8epa annual), 4^th^ W90 (annual), the 4^th^ highest daily maximum 8-h during the summer (4^th^ dma8epa summer), the number of exceedances of daily maximum 8-h values greater than 70 ppb during the summer (nvgt70 summer), and 3-month running mean (i.e., metrics influenced by higher concentrations).
2. SOMO35, SOMO10, and average MDA8epax summer (i.e., metrics influenced by a mixture of low, moderate, and high levels).

Thus, for the human health metrics, the analysis using TOAR data between 1995 and 2014 indicates that groups of metrics influenced by similar ranges of ozone levels within a distribution exhibit trending patterns which are generally consistent with those calculated in the case study (Tables 5 and S-4).

Vegetation metrics fell into three distinct groups:

1. 3-month AOT40 wheat, 3-month 12-h W126 wheat, 3-month 24-h W126 wheat, and 3-month M12 wheat.
2. 6-month AOT40 summer, 6-month W126 12-h summer, 6-month W126 24-h summer, 3-month AOT40 rice, 3-month W126 12-h rice, 3-month W126 24-h rice, and 3-month M12 rice.
3. 6-month M12 summer, 3-month M12 rice, and mean summer.

Because the accumulation period associated with the 3-month period for the vegetation metrics are dependent upon global regions (see Section 2), no attempt was made to identify the ranges most influencing the individual metrics. For the 6-month metrics, a fixed set of months (April–September NH and October–March SH) was applied for the accumulation period and the ranges were identified. Also note that the 3-month wheat growing season tends to occur earlier in the spring compared to a summer rice growing season in most locations. Therefore, the 3-month rice metrics were more likely to have similar trend patterns to 6-month summer metrics than the 3-month wheat metrics.

For the 6-month vegetation metrics, similar conclusions to those from the human health metrics are reached. In most cases, the metrics associated with the moderate and high levels within the distribution influenced the distinct groups and this was generally consistent with those calculated in the case study.

#### 4.2.1. Mean and median concentrations

**Figure 11:**
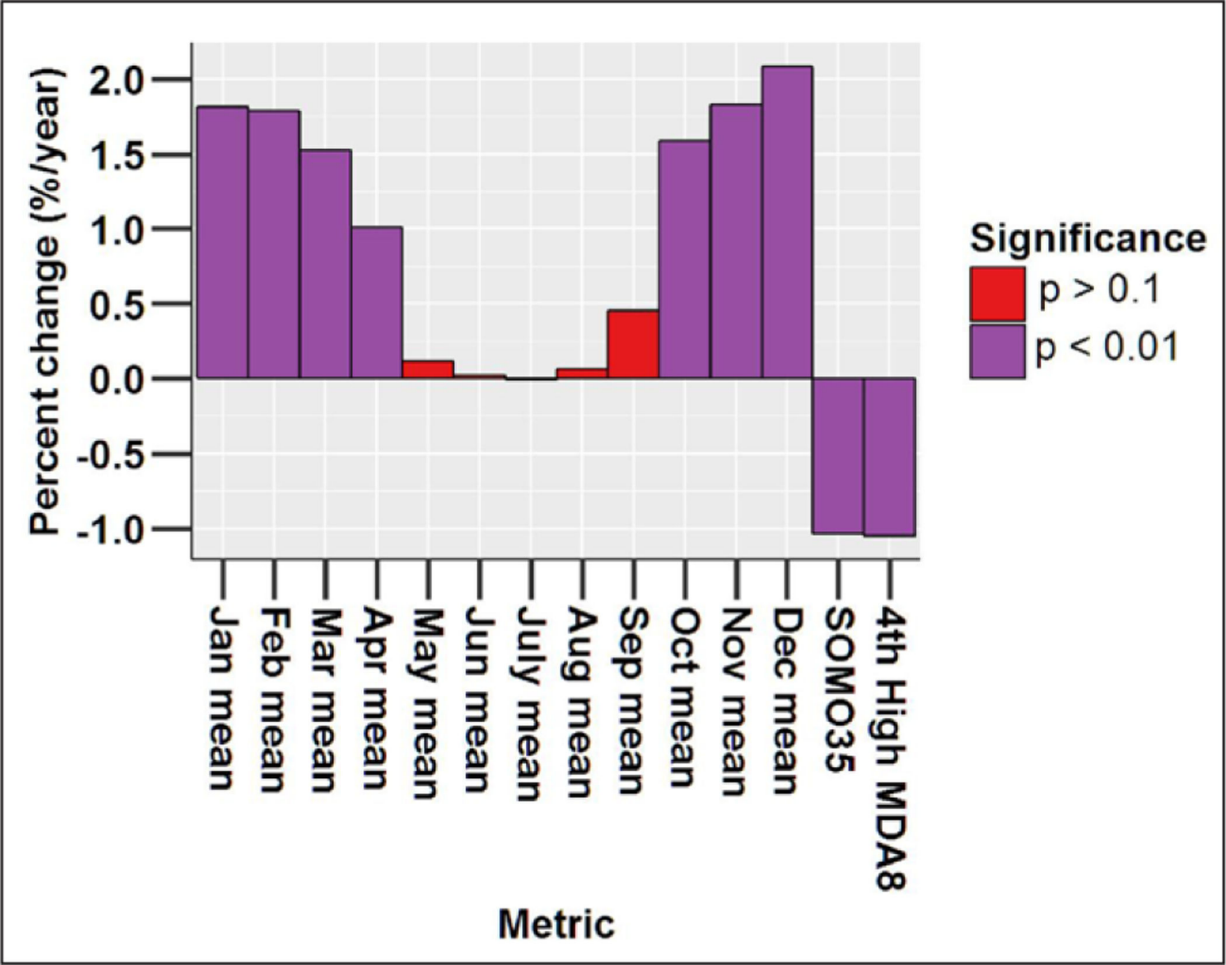
The Theil-Sen (%/year) trend in monthly average ozone levels and the annual SOMO35 and 4 th highest MDA8 human health metrics (A4MDA8) for a suburban site for 1980–2013 in Philadelphia, Pennsylvania (US EPA AQS ID: 421010024–1). The p < 0.05 value was used to determine significance using the Mann-Kendall test. DOI: https://doi.org/10.1525/elementa.279.f11

Table 5 also compares the case study metrics with two metrics that have not been specifically linked to human health or vegetation impacts, but which are often used to characterize ozone trends and to evaluate global models, the annual and summer mean and median hourly ozone concentrations. There are varying levels of agreement between trends in mean and median concentrations versus different metrics relevant to human health and vegetation. Trends in the human health metric impacted by the high end of the distribution bear the least resemblance to trends in the mean and median values with generally less than 50% of sites having trends in the same direction. Of the human health metrics, SOMO10 had the most sites with trends in the same direction as mean and median trends (64%–83% of sites). Depending on the metric, between 39% and 81% of sites had trends in the same direction between the vegetation exposure metrics and mean/median ozone concentration metrics, with the best agreements occurring with the M12 vegetation metrics. Overall, trends in the four mean/median metrics were not representative of the trends behavior of most of the human health and vegetation exposure metrics. Therefore, modeling results indicating increases or decreases in mean or median concentrations may not reflect changes in health or vegetation impacts. In addition, median metrics showed less correspondence with the effects metrics than the mean metrics and the annual mean/median metrics showed less correspondence with the various effects metrics than the summer mean/median values. These findings are consistent with those reported by Lefohn et al. (2017) that trends in mean or median concentrations did not appear to be well associated with some of the exposure metrics applicable for assessing human health or vegetation effects. Figure 11 compares trend patterns for monthly average concentrations (another commonly used metric for global model evaluations), annual SOMO35, and annual 4^th^ highest daily maximum 8-h concentration (A4MDA8) exposure metrics at a suburban site in Philadelphia, Pennsylvania. The monthly average concentrations significantly increased for seven of the 12 months, and were never estimated to decrease, while the SOMO35 and the A4MDA8 metrics significantly decreased.

## 5. Summary and conclusions

A key component of the TOAR project is the consistent calculation of a suite of ozone metrics across thousands of monitoring sites across the globe. Human health and vegetation metrics provide information for assessing spatial and temporal variation in ozone relevant to these impacts. In addition, these metrics, calculated at individual sites, provide insight into the physical and chemical processes that determine ozone and its variations on different time-scales. Comparison of metrics calculated at surface sites to modeled ozone levels is one method used to evaluate the performance of global models in predicting tropospheric ozone. However, owing to different scientific evidence underpinning each metric, different policy considerations, or features of ozone variability that are of interest, multiple metrics with varying forms have been defined to assess ozone relevant for human health and vegetation impacts, and for model-measurement comparison purposes. This paper has provided the necessary information to understand the implications of selecting any one of these metrics for assessing spatial and temporal trends in ozone and describes the scientific rationale associated with the derivation of the metrics.

In addition to the consistent calculation of metrics across monitoring sites, a consistent approach was also required to quantify the magnitude, direction, and statistical significance of long-term trends in these metrics. To achieve this, the nonparametric Mann-Kendall (M-K) test was used to identify significant trends and the Theil-Sen (T-S) statistic to estimate the magnitude of the trend TOAR assessed over the specified period. There was no evidence of worrisome levels of autocorrelation for the annual metrics over the 15-year period (2000–2014) used in TOAR. In the calculation of the TOAR metrics, and the subsequent determination of trends, all hourly averaged ozone data are used subject to the data capture criteria as indicated in the database descriptive materials. TOAR made the decision to generally not combine data from different sites but a few exceptions were made and are noted in the TOAR database.

Hourly ozone values are used to calculate the magnitude, spatial distribution, and trend for various exposure metrics associated with human health, vegetation, and climate. Exposure metric trends are associated with changes in the frequency of hourly average concentrations across an ozone distribution. The results described in this paper underline the sensitivity of different metrics to different patterns of change across ozone distributions. For example, metrics which focus on the highest concentrations, such as A4MDA8, are sensitive to the magnitude of changes occurring predominately only at these peak concentrations, and independent of changes occurring across the rest of the ozone concentration distribution. In contrast, other metrics which focus on a wider range of hourly average concentrations (e.g., SOMO35, W126, AOT40, daily 12-h average, and SOMO10) are determined by the relative magnitude of changes occurring in different parts of the ozone distribution. Consequently, changes in the ozone distribution at a site may result in different trends in the different metrics used to assess human health and vegetation. Thus, understanding the relationship between trends in exposure metrics and ozone distribution changes is essential for predicting or evaluating changes in human health and vegetation metrics that result from the drivers of ozone variability, as well as assessing the effectiveness of control strategies.

## Supplementary Material

Supplemental Information

## Figures and Tables

**Table 1:** Summary of the metrics relevant for model-measurement comparison (Section 2.3.1), characterization of free tropospheric ozone (Section 2.3.2), human health impacts (Section 2.3.3), and vegetation impacts (Section 2.3.4). DOI: https://doi.org/10.1525/elementa.279.t1

Metric	Units	Application Area	Example Refeence(s)
Monthly mean of the 24-h average values	ppb	Model-measurement comparison metrics	Young et al. (2018) and references therein
Monthly standard deviation, median, 5^th^, 25^th^, 75^th^, and 95^th^ percentiles of the maximum daily average 8-h (MDA8) ozone values	ppb	Model-measurement comparison metrics	Fiore et al. (2014); Dolwick et al. (2015)
Monthly mean diurnal cycle (monthly average of 1-h ozone averages at 0100 h, 0200 h, 0300 h, etc.)	ppb	Model-measurement comparison metrics	Schnell et al. (2015)
Monthly mean of daily minimum and maximum hourly average ozone values	ppb	Model-measurement comparison metrics	Schnell et al. (2015)
Monthly, seasonal, annual and decadal means from ozonesonde, aircraft, and lidar measurements on pressure surfaces at intervals of 25 hPa from 1000 hPa to the tropopause. Standard deviations, median and 5^th^, 25^th^, 75^th^, and 95^th^ percentiles are provided where sampling is sufficient.	ppb	Free tropospheric metrics	Young et al. (2018) and references therein
Monthly mean diurnal cycle at hourly intervals with high frequency aircraft data (MOZAIC-IAGOS), and also lidar where data frequency permits.	ppb	Free tropospheric metrics	Young et al. (2018) and references therein
Monthly mean tropospheric column ozone (TCO) from satellite instruments	Dobson Units	Free tropospheric metrics	Young et al. (2018) and references therein
Monthly mean (Total Column Ozone (TCO) from ozonesondes	Dobson Units	Free tropospheric metrics	Young et al. (2018) and references therein
Estimates of the annual cycle, at monthly intervals, averaged over each decade on 25 hPa pressure surfaces or for TCO	ppb/Dobson Units	Free tropospheric metrics	Young et al. (2018) and references therein
The 4^th^ highest MDA8 ozone value over the entire year (see text for specific calculation protocols).	ppb	Human health	US Federal Register (2015)
Maximum daily 8-h average over the entire year	ppb	Human health	European Council Directive 2008/50/EC; WHO (2006); Kamyotra et al. (2012); SANS (2011); Qiao et al. (2015)
Maximum daily 1-h average ozone value over the entire year.	ppb	Human health	European Council Directive 2008/50/EC; Kamyotra etal. (2012); Qiao etal. (2015)
4th highest W90 5-h cumulative exposure index	ppb-hrs	Human health	Lefohn etal. (2010b)
SOMO35: Annual sum of the positive differences between the daily maximum 8-h average ozone value and the cutoff value set at 35 ppb	ppb-day	Human health	Amann et al. (2008); REVIHAAP (2013)
SOM010: Annual sum of the positive differences between the daily maximum 8-h average ozone value and the cutoff value set at 10 ppb	ppb-day	Human health	REVIHAAP(2013)
Number of exceedances of daily maximum 1-h average values greater than 90, 100, and 120 ppb per year	number of hours	Human health	Qiao etal. (2015)
Number of exceedances of daily maximum 8-h average values greater than 50, 60, 70, and 80 ppb per year	number of hours	Human health	US Federal Register (2015); WHO (2006); European Council Directive 2008/50/EC
Running mean of the 3-month average of the daily 1-h maximum ozone value	ppb	Human health	Brauer et al. (2016)
Annual and summertime mean of the daily maximum 8-h average values	ppb	Human health	Turner et al. (2016)
Annual and seasonal percentiles (median, 5th, 25th, 75th and 95th) of all hourly average values.	ppb	Human health	Xu et al. (2008); Simon et al. (2015)
W126 for various months and daily time periods (see text)	ppb-hrs	Vegetation	Lefohn etal. (1988)
AOT40 for various months and daily time periods (see text)	ppb h	Vegetation	CLRTAP(2017)
Daily 12-h average for various months and daily time periods (see text)	ppb	Vegetation	Heck et al. (1988); Jager et al. (1992); Legge et al. (1995)
Seasonal percentiles (median, 5th, 25th, 75th, 95th, 98th, and 99th) of hourly average ozone values	ppb	Vegetation	Xu etal. (2008)
Flux-Based Indices	nmol m^−2^ Projected Leaf Area s^−1^	Vegetation	Emberson et al. (2000); Mills et al. (2011b)

**Table 2:** Comparison of the Mann-Kendall and linear regression applied to annual fourth highest daily maximum 8-h level using data from a site at Harwell, UK. DOI: https://doi.org/10.1525/elementa.279.t2

Method	Time period	Number of valid years	Trend estimate (ppb/yr)	*p*-value	Lower 95% conf. limit (ppb/yr)	Upper 95% conf. limit (ppb/yr)	R^2^ (%)
Mann-Kendall	1984–2013	27	−0.86	0.0005	−1.69	−0.36	NA
regression	1984–2013	27	−0.92	0.0010	−1.42	−0.41	36

**Table 3:** Mann-Kendall and linear regression applied to annual 95^th^ percentile at the Look Rock, TN site. DOI: https://doi.org/10.1525/elementa.279.t3

Method	Time period	Number of valid years	Trend estimate (ppb/yr)	*p*-value	Lower 95%conf. limit (ppb/yr)	Upper 95% conf. limit (ppb/yr)	R^2^ (%)
Mann-Kendall	1990–2013	24	−0.31	0.0866	−0.84	0	NA
regression	1990–2013	24	−0.36	0.0770	−0.77	0.04	14

**Table 4:** List of the 14 exposure metrics from the case study influenced by different portions of the ozone distribution (Lefohn et al., 2017). DOI: https://doi.org/10.1525/elementa.279.t4

Exposure metrics influenced by high hourly average ozone levels	Exposure metrics influenced by moderate and high hourly average ozone levels	Exposure metrics influenced by moderate hourly average ozone levels	Exposure metrics influenced by low, moderate, and high hourly average ozone levels
Annual 4th highest daily maximum of the 8-h ozone level (A4MDA8) based on the US EPA protocol used in the 2008 8-h standard Annual maximum of the daily 8-h ozone level (AmaxMDA8) based on the US EPA protocol used in the 2008 8-h standard Annual maximum daily 1-h average level (AmaxMDAl) 4th highest W90 (A4W90)	SOMO35 12-h W126 3-month 12-hW126 6-month 24-h W126 3-month 24-h W126 6-month 12-h AOT40 3-month 12-h AOT40 6-month	Daily 12-h average (M12) averaged over 3-months Daily 12-h average (M12) averaged over 6-months	SOMO10

**Table 5:** Percentage of sites included in TOAR for which the trends (from 1995 through 2014) in TOAR exposure metrics (columns) were in the same direction (i.e., decreasing, increasing, or no significant change) compared to a set of metrics (rows), which included those in Lefohn et al. (2017). The mean and median metrics are included for comparative purposes.* DOI: https://doi.org/10.1525/elementa.279.t5

	4th dma8epa summer	nvgt070 summer	3-month running mean	average MDA8 epax summer	3-month aot40 rice	3-month w126_12h rice	3-month w126_24h rice	3-month M12 rice	median annual	mean annual	median summer	mean summer
**4th W90**	92%	83%	80%	67%	75%	80%	80%	63%	32%	38%	41%	48%
**4th dma8epa**	95%	84%	82%	70%	78%	80%	79%	65%	33%	39%	43%	50%
**SOMO35**	69%	74%	75%	86%	73%	71%	71%	80%	49%	57%	62%	71%
**SOM010**	55%	61%	61%	77%	62%	59%	59%	76%	64%	74%	76%	83%
**3-month aot40 wheat**	58%	63%	65%	88%	66%	62%	62%	69%	56%	64%	65%	72%
**6-month aot40 summer**	79%	84%	80%	75%	83%	81%	82%	81%	43%	51%	51%	62%
**3-month wl26_12h wheat**	65%	68%	71%	77%	72%	67%	69%	69%	51%	59%	60%	70%
**6-month wl26_12h summer**	83%	88%	82%	83%	86%	85%	85%	82%	39%	46%	46%	57%
**3-month wl26_24h wheat**	62%	65%	69%	75%	69%	63%	65%	70%	53%	61%	61%	70%
**6-month wl26_24h summer**	82%	86%	82%	85%	84%	82%	83%	81%	41%	48%	48%	59%
**3-month M12 wheat**	45%	48%	52%	65%	51%	49%	49%	58%	69%	74%	76%	77%
**6-month M12 summer**	60%	66%	66%	87%	71%	67%	68%	85%	56%	66%	70%	81%
**4th dma8epa summer**	100%	87%	84%	72%	80%	83%	82%	68%	33%	40%	39%	48%
**nvgt070 summer**	87%	100%	83%	77%	86%	87%	88%	78%	37%	44%	43%	53%
**3-month running mean**	84%	83%	100%	76%	77%	79%	79%	65%	37%	44%	43%	53%
**average MDA8 epax summer**	72%	77%	76%	100%	79%	76%	78%	88%	48%	58%	59%	71%
**3-month aot40 rice**	80%	86%	77%	79%	100%	94%	93%	82%	39%	46%	44%	53%
**3-month wl26_12h rice**	83%	87%	79%	76%	94%	100%	96%	77%	35%	43%	41%	50%
**3-month wl26_24h rice**	82%	88%	79%	78%	93%	96%	100%	79%	38%	44%	43%	51%
**3-month M12 rice**	68%	78%	65%	88%	82%	77%	79%	100%	54%	60%	60%	68%

* For two of the TOAR exposure metrics (i.e., AmaxMDA8 and AmaxMDA1) analyzed in Lefohn et al. (2017), trend analyses were not available in the TOAR preformatted files.
