# Supplementary material for: Tropospheric ozone assessment report: Global ozone metrics for climate change, human health, and crop/ecosystem research
Source: Elementa (Wash D C). Author manuscript; Available in PMC 2018 Oct 17. (PMC6192432; doi:10.1525/elementa.279)
Supplement: Supplemental Information [file NIHMS990043-supplement-Supplemental_Information.pdf]

List of Contents:

Additional Metrics Derived from Long-term Observations at Baseline Monitoring Sites (Pages 2-5)

Detailed Description of TOAR Human Health Metrics (Pages 6-14)

Detailed Description of TOAR Vegetation Metrics (Pages 14-19)

Table S-4: Percentage of sites included in TOAR for which the trends (from 1995 through 2014) in each metric from Lefohn et al. (2016) were in the same direction as all other exposure metrics from Lefohn et al. (2017). (Page 20).

Additional References Cited in Supplement Not Listed in the Main Paper (Pages 21-23)

Additional Metrics Derived from Long-term Observations at Baseline Monitoring Sites

Polynomial "shape factors" that describe long-term trends are calculated for selected baseline sites and made available in table form, as described by Parrish et al. (2014). A systematic approach to quantifying long-term changes in baseline ozone, based upon the available data sets collected at relatively isolated sites from the mid-twentieth century to the present yielded metrics, was applied to represent long-term changes in seasonally averaged baseline ozone levels at northern mid-latitudes suitable for evaluating the analogous ozone trends calculated by models (Parrish et al., 2014). The metrics themselves are the coefficients of polynomial fits to the measured levels following normalization to the year 2000 intercepts of the data. The intercepts (Table S-1) provide metrics for comparing absolute ozone levels at specific locations. The levels in Table S-1 should be interpreted as the seasonally averaged, near surface, baseline ozone level in the year 2000 with the interannual variability removed. Thus, these metrics are suitable for comparison to both Coupled Climate Models (CCMs), which simulate their own meteorology that is not intended to reproduce the actual meteorological variability, and chemical transport models, which assimilate real meteorology. The polynomial coefficients (Table S-2) provide metrics that characterize relative ozone level changes over broad regions of the northern mid-latitude lower troposphere. These polynomial coefficients define the black curves illustrated in Figures 5 and 6 of Section 6, *Model Performance*).

Fourier series expansion of seasonal cycles at selected baseline sites are calculated for selected baseline sites and made available in the TOAR database. Fourier series expansions of seasonal cycles of ozone at marine boundary layer (MBL) sites around the globe have been shown to provide critical tests of the model treatment of some of the physical processes that control tropospheric ozone levels (Parrish et al., 2015). The annual average plus two sine function terms - the fundamental (period = 1 year) and second harmonic (period = 1/2 year) - are the only significant contributors to the seasonal cycles of ozone at these sites. Figure 1 in the paper illustrates one example. Thus, the seasonal cycle can be defined by:

$$y = Y_o + A_1 * sin(\chi + \phi_1) + A_2 * sin(2 * \chi + \phi_2). \quad (1)$$

Here the variable χ spans one year's time period in radians from 0 to 2π. The five parameters of these three terms (Table S-3) provide metrics to which model calculations can be quantitatively compared: Y_0 (the annual average) and the amplitudes (A_1, A_2) and phases (ϕ_1, ϕ_2) of the two sine terms. An additional metric is the root-mean-square deviation (RMSD) of the monthly average from the functional form of Equation (1); this metric is also included in Table S-3.

Table S-1: Year 2000 intercepts of quadratic fits to three-month seasonally averaged ozone level measurements at relatively remote (baseline) sites at northern mid-latitudes. Levels are given as mixing ratios in ppb. 95% confidence limits are indicated.

Site	MAM	JJA	SON	DJF
Arkona-Zingst, Germany	34.9 ± 1.3	33.6 ± 1.7	21.8 ± 1.0	20.6 ± 1.1
Mace Head, Ireland	44.4 ± 1.1	34.8 ± 1.1	36.4 ± 1.1	42.4 ± 0.8
Hohenpeissenberg, Germany	45.9 ± 1.3	50.1 ± 2.0	31.8 ± 1.0	31.9 ± 1.1
Arosa, Switzerland	48.6 ± 1.2	46.7 ± 1.6	36.2 ± 1.1	36.4 ± 1.3
Zugspitze, Germany	56.4 ± 1.8	58.5 ± 1.4	46.0 ± 1.0	44.5 ± 1.2
Jungfraujoch, Switzerland	57.2 ± 1.6	59.4 ± 1.9	48.7 ± 0.8	46.0 ± 1.1
European alpine[a]	56.6 ± 0.9	58.3 ± 0.9	46.6 ± 0.6	44.9 ± 0.6
U.S. Pacific Coast MBL	39.2 ± 0.9	27.6 ± 1.8	30.9 ± 1.2	33.6 ± 1.5
Lassen NP California U.S.	42.6 ± 1.3	45.5 ± 1.9	38.8 ± 1.6	38.4 ± 1.5
North American FT*	60.7 ± 1.3	----	----	----
Japanese MBL	54.0 ± 1.3	37.3 ± 2.4	40.1 ± 2.1	42.3 ± 2.5
Mt. Happo, Japan	65.4 ± 1.3	50.4 ± 3.0	48.0 ± 2.1	48.5 ± 2.4

a This dataset combines the results from three alpine sites in Europe (Zugspitze, Germany; Sonnblick, Austria and Jungfraujoch, Switzerland), all of which exhibit very similar ozone levels.

* North American Free Troposphere.

Table S-2: Coefficients of polynomial fits that define the shape factors derived from the seasonal average measurements. The fits are of the form $y = a + bt + ct^2 + dt^3 + et^4$ where t is in years since 2000, and the coefficients in the table have been divided by the factor indicated below the respective coefficient symbol. Units of coefficients are those required so that each term in the polynomial gives a dimensionless number. 95% confidence limits are indicated.

Season	a	b	c ($x10^{-2}$)	d ($x10^{-4}$)	e ($x10^{-6}$)	Years of Fit
			European alpine[a]			
MAM	99.9 ± 1.1	0.24 ± 0.15	-4.5 ± 1.2	---	---	1998-2011
JJA	99.6 ± 1.4	-0.12 ± 0.19	-5.5 ± 1.5	---	---	1998-2011
SON	99.3 ± 1.2	0.16 ± 0.16	-3.7 ± 1.2	---	---	1998-2011
DJF	99.3 ± 1.2	0.56 ± 0.17	-4.7 ± 1.3	---	---	1999-2011
			Europe			
MAM	100.5 ± 1.5	0.43 ± 0.18	-4.1 ± 1.6	-5.7 ± 3.2	---	1951-2010
JJA	101.1 ± 2.0	0.04 ± 0.21	-6.2 ± 2.6	-11.3 ± 9.5	-5.4 ± 9.5	1934-2010
SON	99.2 ± 1.7	0.36 ± 0.21	-1.5 ± 1.8	-0.1 ± 3.5	---	1950-2010
DJF	99.4 ± 1.8	0.82 ± 0.22	-2. 8 ± 1.9	-3.9 ± 3.7	---	1950-2010
			North America and Asia			
MAM	99.6 ± 1.4	0.93 ± 0.16	-2.2 ± 2.2	---	---	1984-2011
JJA	99.8 ± 2.8	0.85 ± 0.27	-6.4 ± 4.4	---	---	1988-2011
SON	99.9 ± 2.4	0.39 ± 0.25	-3.2 ± 4.1	---	---	1988-2011
DJF	99.3 ± 2.2	0.92 ± 0.23	-2.6 ± 3.9	---	---	1988-2011

[a] This dataset combines the results from three alpine sites in Europe (Zugspitze, Germany; Sonnblick, Austria and Jungfraujoch, Switzerland).

Table S-3: Parameters of Fourier Series fits to ozone seasonal cycles for 11 MBL surface data sets. 95% confidence limits are indicated.

Monitoring Site	Y_o[a] (ppb)	A_1 (ppb)	ϕ_1 (radians)	A_2 (ppb)	ϕ_2 (radians)	RMSD[b] (ppb)
Arrival Heights, Antarctica	25.7 ± 0.5	9.4 ± 0.8	-1.93 ± 0.08	0.9 ± 0.8	-2.10 ± 0.89	[illegible].5
Ushuaia, Argentina	23.7 ± 0.4	9.0 ± 0.5	-1.97 ± 0.06	1.1 ± 0.5	-2.08 ± 0.47	[illegible].3
Cape Grim, Australia	25.0 ± 0.2	7.1 ± 0.2	-2.10 ± 0.03	1.7 ± 0.2	-1.77 ± 0.14	[illegible].1
Cape Point, South Africa	23.1 ± 0.4	7.2 ± 0.6	-2.09 ± 0.08	0.9 ± 0.6	-2.07 ± 0.60	[illegible].5
Cape Matatula, Samoa	13.5 ± 0.3	5.4 ± 0.4	-2.31 ± 0.08	1.0 ± 0.4	1.59 ± 0.41	[illegible].4
Ragged Point, Barbados	21.2 ± 1.2	5.3 ± 1.7	0.91 ± 0.32	1.1 ± 1.7	1.35 ± 1.55	[illegible].6
Tudor Hill, Bermuda	37.3 ± 1.3	10.3 ± 1.9	0.67 ± 0.18	5.3 ± 1.9	-2.32 ± 0.36	[illegible].4
Japanese MBL	45.1 ± 1.4	8.3 ± 2.0	-0.04 ± 0.24	5.9 ± 2.0	-2.31 ± 0.34	[illegible].4
Pacific MBL, USA	32.0 ± 0.7	5.7 ± 0.9	0.48 ± 0.16	3.5 ± 0.9	-2.30 ± 0.26	[illegible].2
Mace Head, Ireland	38.8 ± 0.4	5.6 ± 0.6	0.50 ± 0.10	3.0 ± 0.6	-2.34 ± 0.20	[illegible].5
Storhofdi, Iceland	38.5 ± 0.5	6.2 ± 0.7	0.53 ± 0.12	3.2 ± 0.7	-2.24 ± 0.23	[illegible].1

[a]Y_o represents the annual average ozone level evaluated over the complete period covered by the measurements.

[b]RMSD is the root-mean-square deviation of the monthly average data from the Fourier Series fits.

Detailed Description of TOAR Human Health Metrics

The procedure for the calculation of the metrics (including data completeness and rounding) is described in Schutz et al. (2017).

a) *4th Highest 8-h Average Concentration for Each Year. Twenty-four running 8-h averages are used to identify the daily maximum 8-h value. The 8-h running mean for a particular hour is calculated on the concentration for that hour plus the following 7 hours.* This form of the metric is based on the US EPA protocol used in the 2008 8-h standard (US EPA, 2014b).

The US EPA's ozone NAAQS was set in 1979 to 0.12 ppm (1-h averaging time, not be exceeded more than 1 day per year over a three-year period), and subsequently revised to 0.08 ppm in 1997 and 0.075 ppm in 2008 (8-h averaging time, 4th highest daily maximum averaged over 3 years) (http://www.epa.gov/ttn/naaqs/standards/ozone/s_o3_history.html). The rationale for the selection of the annual 4th highest daily maximum 8-h average versus the 2nd, 3rd, or 5th value is discussed by the US EPA in the US Federal Register (1997). For calculating the 8-h daily values for the 2008 8-h standard of 0.075 ppm, there are 24 8-h periods that start in each calendar day. The daily maximum 8-h concentration for a given calendar day is the highest of the 8-h average concentrations computed for 8-hour periods that start in that day. This process is repeated, yielding a daily maximum 8-hour average ozone concentration for each day with ambient ozone monitoring data, including days outside the required ozone monitoring season if data are available. The daily maximum 8-h concentrations from two consecutive days may have some hourly concentrations in common. Generally, overlapping daily maximum 8-h averages are not likely, except in those non-urban monitoring locations with less pronounced diurnal variation in hourly concentrations or with a pronounced diurnal pattern that peaks at night due to transport lag time. In these cases, the maximum 8-h average concentration from each day is used, even if the two averages have some hours in common.

b) 4th Highest 8-h Average Concentration for Each Year. Twenty-four running 8-h averages are used to identify the daily maximum 8-h value. Using the procedures specified in the EU Airbase, the 8-h running mean is determined based on the concentration for that hour and the previous 7 hours.

The calculation of the 24 daily running 8-h ozone concentrations specified in EU AirBase documentation (Garber et al., 2002; European Council Directive 2008/50/EC) differs from the US EPA protocol (US EPA, 2006), as the 8-h running mean for a particular hour is based on the ozone concentration for that hour and the previous 7 hours. This means that, for example, the 8-h running mean for the hour starting at 00:00 on a particular day using the EU method (preceding hours), will be calculated using the same 8 hourly ozone concentrations as the 8-h running mean for the hour starting at 17:00 on the

previous day calculated using the US EPA method (following hours). This method for calculation of 8-h running means has also been adopted elsewhere, e.g. in South African air quality standards (SANS, 2011). Hence on a particular day, 7 of the 24 running 8-h ozone concentrations used to calculate the daily maximum 8-h ozone concentration will be different for the two methods. By including both the EU and the US EPA protocols, a comparison is possible for the two methods. Previous application of the 4th highest 8-h average concentration metric to ozone measurements at European monitoring stations applied the EU protocol to derive the 8-h running means (Tripathi et al., 2012).

c) *4th Highest 8-h Average Concentration for Each Year*. The form of this metric is based on the US EPA protocol adopted on 1 October 2015 (US Federal Register, 2015).

The US EPA's ozone NAAQS was changed from 0.075 ppm to 0.070 ppm (8-h averaging time, fourth highest daily maximum averaged over 3 years) on 1 October 2015. The US EPA noted that compared to (1) estimates of ozone exposures of concern and (2) estimates of ozone-induced lung function decrements, the Agency's conclusions concerning the level of the standard reflected lower confidence in epidemiologic-based risk estimates (US EPA, 2015). In establishing the new ozone standard, the US EPA indicated that it was confident that reducing the highest ambient ozone concentrations would result in substantial improvements in public health, including reducing the risk of ozone-associated mortality. The Agency noted that it was far less certain about the public health implications of the changes in relatively low ambient ozone concentrations. The Agency therefore concluded that reducing precursor emissions to meet the new lower ozone standard would result in important reductions in ozone concentrations from the highest part of the air quality distribution, where the scientific evidence provided the strongest support for adverse health effects.

Unlike the form of the 2008 ozone standard (0.075 ppm 8-h average), the daily maximum 8-h average ozone concentration for a given day is derived from the highest of the 17 consecutive 8-h averages beginning with the 8-h period from 7:00 a.m. to 3:00 p.m. and ending with the 8-h period from 11:00 p.m. to 7:00 a.m. the following day (i.e., the continuous 8-h averages running from 7:00 a.m. to 11:00 p.m.). The daily maximum 8-h average ozone concentration is determined for each day with ambient ozone monitoring data, including days outside the ozone monitoring season if those data are available. This method ensures that the MDA8 on two consecutive days will not have any hours in common and prevents double counting of high overnight ozone events.

d) *Maximum daily 8-h average over the entire year*.

For each day in a year, the maximum of the 24 8-h running means is calculated according to the EU protocol, and the highest of these values constitutes the

maximum daily 8-h average over the entire year. The annual maximum daily 8-h average ozone value is the basis for the EU ozone long term objective for the protection of human health, set at 120 $\mu g\ m^{-3}$ (60 ppb) (European Council Directive 2008/50/EC). This metric is calculated for ozone data at all European sites archived in the EU AirBase data repository (http://www.eea.europa.eu/data-and-maps/data/aqereporting), and it has been used previously to assess temporal and spatial variation in health-relevant ozone across Europe (EEA, 2014a; EEA, 2014b; Guerreiro et al., 2014; Tripathi et al., 2012). This metric emphasizes the magnitude of the high percentile ozone concentrations occurring during a given year, which generally result from regional or local photochemical episodes, rather than baseline concentrations (Royal Society, 2008). Additionally, this metric can be combined with another metric, the number of exceedances of daily maximum 8-h ozone values greater than 60 ppb (outlined below), to understand both the magnitude of exceedance of the EU long term ozone objective, and the frequency with which exceedance occurs. Other international air quality guidelines and standards are also based on maximum daily 8-h average values, including the WHO air quality guideline (set at 100 $\mu g/m^3$ (50 ppb)), (WHO, 2006)), and air quality standards in India (50 ppb, (Kamyotra et al., 2012)), South Africa (60 ppb, (SANS, 2011) and China (75 ppb, (Qiao et al., 2015)). Both the EU directive and WHO use limit values given in $\mu g/m^3$, which is approximated by ppb×2.

e) Maximum daily 1-h average over the entire year.

The annual maximum daily 1-h average ozone concentration is the highest 1-h ozone concentration measured at a site across the year. This metric can be used to compare health-relevant ozone at a site against the EU 'information threshold', set at 180 $\mu g\ m^{-3}$ (90 ppb). The directive also has an alert threshold of 240 $\mu g/m^3$. The EU information thresholds have been established as hourly ozone concentrations 'beyond which there is a risk to human health from brief exposure for particularly sensitive sections of the population', and about which the public must be informed (European Council Directive 2008/50/EC). Previous studies at sites have used the information threshold as a basis for assessment of temporal and spatial variation in short-term peak ozone (EEA, 2014a; Jenkin, 2008). This metric quantifies the highest hourly-average ozone concentration and does not provide any information about the distribution of ozone concentrations below the peak value. However, this metric can be combined with another metric, the number of exceedances of daily maximum 1-h ozone concentrations greater than 90 ppb (outlined below), to understand both the magnitude of maximum exceedance of the EU information threshold, and the frequency with which exceedance occurs. Other international air quality standards are based on the maximum hourly value, including those established in India (90 ppb, (Kamyotra et al., 2012)), China (93 ppb, (Qiao et al., 2015)), Australia (100 ppb, (EPA Victoria, 2014)) and Japan (60 ppb, (de Leeuw and Ruyssenaars, 2011)).

f) SOMO35(i.e., the annual sum of the positive differences between the daily maximum 8-h ozone average value and the cutoff value set at 35 ppb (70

µg/m³) calculated for all days in a year). The 8-h average values are determined as per Item (b) above.

The Sum Of Means Over 35 ppb (SOMO35) metric is the annual sum of the positive differences between the daily maximum 8-h average ozone concentrations, and the cutoff concentration set at 35 ppb (70 μg m^{-3}), calculated for all days in a year. The SOMO35 was developed and applied in Amann et al. (2008), based on the recommendation of a 35 ppb (70 μg m^{-3}) cut off for integrated assessment modelling. The ozone concentration selected as the cutoff was chosen partly due to the more accurate modeled ozone concentrations available above 35 ppb, but also due to the observation of a statistically significant increase in mortality calculated for short-term exposure to ozone values above 25-35 ppb (Gryparis et al., 2004).

Subsequently, the World Health Organization (WHO) Review of Evidence on the Health Aspects of Air Pollution (REVIHAAP, 2013) synthesis report provided recommendations for the quantification of ozone relevant for health effects associated with short-term exposure which are in line with those used to calculate SOMO35 (and SOMO10, see below). Specifically, the SOMO35 metric is accumulated based on daily maximum 8-h ozone concentration over the whole year. Association between daily maximum 8-h ozone and mortality have been calculated in winter as well as summer based on analysis for 23 European cities (Gryparis et al., 2004), and in 21 East Asian cities (Chen et al., 2014). Calculation of SOMO35 also assumes a linear concentration-response relationship, which has been calculated in a range of epidemiological studies (Atkinson et al., 2012; Bell and Dominici, 2008; Gryparis et al., 2004). However, other studies have reached different conclusions on the suitability of a linear concentration-response function (Stylianou and Nicolich, 2009). Finally, REVIHAAP also specified two cutoff values set at 10 ppb (20 μg m^{-3}, see SOMO10 metric description below) and 35 ppb (70 μg m^{-3}), as 'the epidemiological evidence on linearity does not extend down to zero'. The recommendation of two cutoffs reflected the 'not consistent' evidence for a threshold for short term exposure and is similar to no effect levels reported in several epidemiological studies (Gryparis et al., 2004; Pattenden et al., 2010). The SOMO35 metric is a standard statistic calculated on ozone time series archived within the EU AirBase data repository. The magnitude of the SOMO35 metric is determined by both the magnitude of ozone values (above 35 ppb) measured at a site, and the frequency with which these values occur.

g) *SOMO10 (i.e., the annual sum of the positive differences between the daily maximum 8-h average ozone value and the cutoff value set at 10 ppb (20 µg/m³) calculated for all days in a year). The 8-h average values are determined as per Item (b) above.*

The Sum Of Means Over 10 ppb (SOMO10) metric is the annual sum of the positive differences between the daily maximum 8-h average ozone values, and the cutoff values set at 10 ppb (10 μg m^{-3}), calculated for all days in a year. The

SOMO10 metric is calculated in the same way as SOMO35, but with the lower cutoff concentration, and reflects the epidemiological evidence of associations between short-term ozone exposure and lower ozone concentrations, as summarized in REVIHAAP (2013). For example, associations between ozone observations and mortality have been calculated at values as low as 10 ppb (Bell and Dominici, 2008; Kim et al., 2004; Stylianou and Nicolich, 2009). Similarly, Bell et al. (2006) concluded that 'a "safe" ozone level can only exist at very low concentrations'. Hence SOMO10 quantifies health-relevant ozone across a larger range of the ozone distribution compared to SOMO35.

Both the SOMO10 and SOMO35 metrics attempt to quantify short-term health-relevant ozone across the range of concentrations which contribute to this impact. The magnitude of each metric will be determined by the trends occurring across the ozone distribution above 10 ppb (SOMO10) and 35 ppb (SOMO35), respectively. Hence opposing trends occurring in different parts of the ozone distribution will act to change the magnitude of SOMO10/SOMO35 in opposite directions. For example, if a decrease in relatively high percentile ozone concentrations coincides with an increase in relatively low ozone concentrations, the magnitudes of SOMO10 and SOMO35 estimate the overall change in ozone exposures resulting from both of these trends. The two cutoff levels represent the inconsistent evidence of the ozone concentration-response relationship for health effects associated with short-term exposure.

h) 4th highest W90 5-h cumulative exposure index as described in Lefohn et al. (2010b).

Controlled human laboratory studies have shown that there is a disproportionately greater pulmonary function response from higher hourly average ozone concentrations than from lower hourly average values and thus, a nonlinear relationship exists between ozone dose and pulmonary function (FEV_1) response (Hazucha and Lefohn, 2007; Lefohn et al., 2010b; US Federal Register, 2015). Lefohn et al. (2010b) reanalyzed data from five controlled human response to ozone health laboratory experiments as reported by Hazucha et al. (1992), Adams (2003, 2006a, 2006b), and Schelegle et al. (2009). These investigators exposed subjects (healthy young adults) to multi-hour variable/stepwise ozone concentration profiles that mimicked typical diurnal patterns of ambient ozone concentrations. Lefohn et al. (2010b) reported a common response pattern across most of the studies that provided information for the development of a lung function (FEV_1)-based 4th highest W90 5-h cumulative exposure index. Based on the reanalysis of the realistic exposure profiles used in these experiments, an alternative form of the human health 8-h standard, similar to the W126 exposure index (Lefohn et al., 1988) that the US EPA currently uses to assess vegetation effects was proposed. The W90 exposure index assigns lower weights to the hourly average values at and below 50 ppb lower than the W126 metric.

The form of the W90 index is $\Sigma\, w_i \times C_i$ with weight $w_i = 1/[1 + M \times exp\,(-A \times C_i/1000)]$, where $M = 1400$, $A = 90$, and where C_i is the hourly average ozone mixing ratio in units of ppb. The M and A constants were derived based on the observations noted in Lefohn et al. (2010b). The W90 index has units of ppb-hrs. The W90 metric is a non-threshold index that is described as the sigmoidally weighted sum of all hourly ozone values, where each hourly ozone mixing ratio is given a weight that increases from zero to one with increasing concentration. The W90 metric is based on the range of typical hourly average values experienced under ambient conditions.

i) *The annual and seasonal percentiles (median, 5th, 25th, 75th and 95th) of hourly average concentrations over 24-h period.*

Ozone is influenced by multiple factors (e.g., precursor concentrations, local meteorology, transport of air masses, deposition, etc.). These factors determine the production, loss and transport of ozone, and hence the concentration and frequency distribution of ozone at any site. Changes in precursor emissions, meteorology, transport, etc., can cause changes in the frequency distribution of ozone concentration. Therefore, investigation into the spatial and temporal changes in the frequency distribution of ozone concentration can assist in identifying the reasons hourly average concentrations at certain sites change. The percentiles (median, 5th, 25th, 75th and 95th) of hourly average ozone concentrations over a 24-h period are statistics which summarize ozone concentrations for which a given percentage (50%, 5%, 25%, 75%, and 95%) of hourly average ozone concentrations are below. Such statistics can be used as ozone metrics for assessing impacts on human health, vegetation, and climate change. These metrics can be calculated separately from hourly averaged ozone observations at different time intervals. For example, in the TOAR assessment, annual and seasonal ozone observations are calculated.

Annual and seasonal percentiles (median, 5th, 25th, 75th and 95th) summarize ozone concentration at five key points across the frequency distributions of hourly average ozone concentrations within a year and a season, respectively. Long-term changes in these percentile metrics facilitate the assessment of the impacts of the ozone level associated with different factors, such as changes of emission, global climate, long-range transport, etc. (Lefohn et al., 2010b). Long-term changes in the concentrations of ozone precursors can cause trends for different parts of frequency distribution of ozone concentrations (Lefohn et al., 1998, 2010; Brönnimann et al., 2002; Xu et al., 2008; Simon et al., 2015), which are not necessarily consistent. Therefore, studying the long-term variations of ozone using these percentile metrics can help to avoid potential misinterpretation in the cause analysis using single summary statistics (e.g., the mean ozone concentration). As indicated in Section 1, shifting distributions affect human health and vegetation metrics. The shifting that occurs among the hourly average concentrations can result from increased or reduced NO_x titration, as well as changes in background ozone mixing ratios (Lefohn and Cooper, 2015). Many

studies indicate that the responses of human health and vegetation injury and damage to ozone exposure are often nonlinear (Hazucha and Lefohn, 2007; Kickert and Krupa, 1991; Lefohn et al., 1988; Massman et al., 2000) with the result that different weighting factors of ozone hourly average concentrations should be applied. Seasonal and annual percentile ozone metrics provide important data for the weighting in the exposure-response studies.

j) *Number of exceedances of daily maximum 8-h values greater than 50, 60, 70, and 80 ppb per year. The 8-h average values are determined as per Item (c) above.*

Numbers of exceedances of daily maximum 8-h values (using method (c) greater than 50, 60, 70, and 80 ppb per year indicate yearly non-attainment occurrences if the threshold values for daily maximum 8-h ozone standard are 50, 60, 70, and 80 ppb, respectively. The exposure-response relationship for human health impact of ozone is nonlinear and the duration of ozone exposure should be considered in health protection (Lefohn and Foley, 1993; Lefohn et al., 2010b; Rombout et al., 1986). There is no unified ozone air quality standard for every nation. For example, the current daily maximum 8-h ozone standard in the United States is 70 ppb (US Federal Register, 2015) and 75 ppb in China (Ministry of Environmental Protection of the People's Republic of China, 2012), and 60 ppb in Europe (http://ec.europa.eu/environment/air/quality/standards.htm). The WHO has established the ozone air quality guideline and interim target 1 level (daily maximum 8-h mean) at about 50 ppb and 80 ppb, respectively (WHO, 2006). In the TOAR assessment, numbers of exceedances of daily maximum 8-h values greater than 50, 60, 70, and 80 ppb per year are calculated as ozone metrics of human health impact.

k) *Number of exceedances of daily maximum 1-h values greater than 90, 100, and 120 ppb.*

Numbers of exceedances of daily maximum 1-h values greater than 90, 100, and 120 ppb per year indicate yearly non-attainment occurrences if the threshold values for daily maximum 1-h ozone standard are 90, 100, and 120 ppb, respectively. In health effect studies, the 1-h average is often used in the parameterization of exposure-response (Hazucha and Lefohn, 2007). There has been no evidence of a population threshold for ozone, below which no effect is measurable. However, it is clear that higher ozone hourly average concentrations cause greater physiological responses (US EPA, 2013). Therefore, lowering the daily maximum hourly ozone level is one of the ways to reduce the adverse effects of ozone on human health. The daily maximum 1-h values were widely used as an ozone air quality standard before the daily maximum 8-h values were introduced. In 1979, the US EPA adopted the daily maximum 1-h value of 120 ppb as an air quality standard for ozone. This 1-h standard was revoked in 2005 by EPA, but some areas have continued obligations under this standard (https://www.epa.gov/criteria-air-pollutants/naaqs-table). In some countries, the daily maximum 1-h value is still used as the ozone standard. For example, Japan has been using the daily maximum 1-h value of

60 ppb as an ozone standard (http://www.env.go.jp/en/air/aq/aq.html). China is using both the daily maximum 8-h value (75 ppb) and the daily maximum 1-h value (93 ppb) as ozone standards for both residential and commercial areas.

l) Running mean of the 3-month average of the daily 1-h maximum.

Short-term increases in ozone have been linked to a wide array of health responses, including increases in daily mortality (Thurston and Ito, 2001; Bell et al., 2007; Bell et al., 2004). Studies of the impacts of chronic exposure, which are generally thought to have the greatest population health impact, are less common. One of the most influential studies of the impact of chronic ozone exposure on mortality is an analysis of the American Cancer Society (Cancer Prevention Study II) cohort population of nearly 500,000 individuals residing in 96 Metropolitan Statistical Areas across the U.S (Jerrett et al., 2009). This study identified ozone as a risk factor for mortality from respiratory diseases that was independent from co-exposure to particulate matter. The quantitative relationship between ozone exposure and mortality from this study is the basis for the estimates of disease burden attributable to long-term ozone exposure in the Global Burden of Disease (GBD) analyses (Lim et al., 2012; Forouzanfar et al., 2015).

Jerrett et al. (2009) evaluated the risk of mortality associated with the averages of the second (April through June) and third (July through September) quarters of the year. Since the ozone (summer) season varies throughout the globe, for estimation of ozone's contribution to global disease burden (Lim et al., 2012; Forouzanfar et al., 2015), a running 3-month average (of daily 1-h maximum values) was calculated for each (0.1 x 0.1) grid cell over a full year and the maximum of these values was selected to best approximate a location-specific seasonal ozone average that conformed with the above epidemiologic analyses of chronic ozone exposure impacts on mortality (Brauer et al., 2012, 2016). Results from the ACS-CPS-II analyses have been widely applied to quantify premature deaths associated with long-term ozone exposure (Anenberg et al., 2010; Forouzanfar et al., 2015; Lim et al., 2012; Shindell et al., 2016). The TOAR long-term trend results (1995-2014) indicate that this human health metric appears to be more associated with the higher hourly concentrations within the distribution than those values associated with the entire distribution (see Section 4). Coupled with this metric, TOAR reports the day of the year on which the 3-month maximum metric reaches its maximum value.

m) Annual and summertime mean of the daily maximum 8-h values. The 8-h average concentrations are determined as per Item (c) above.

The annual and summertime mean of the daily maximum 8-h values (in units of ppb) are metrics that were used as an estimate of long-term ozone exposure in an epidemiological analysis (Turner et al., 2016). Turner et al. (2016) calculated significant association between annual and summertime daily maximum 8-h ozone concentrations, and all-cause, respiratory, and circulatory mortality within the American Cancer Society Cancer Prevention Study-II (ACS CPS-II) cohort population. This updated analysis of the ACS CPS-II cohort builds on an earlier analysis (i.e., Jerrett et al., 2009), with over twice as

many deaths with which to evaluate the relationships with long-term ozone exposure. The annual metric was selected for consistency with the exposure estimates for PM2.5 and NO_2 in Turner et al. (2016) and the summertime (April-September) average daily max 8-h value was also used for consistency with the time period used to characterize long-term ozone exposure in the previous Jerrett et al. (2009) analysis.

Detailed Description of TOAR Vegetation Metrics

a-h) W126

In 1985, A.S. Lefohn developed the form for the W126 ozone exposure index as an ozone metric that was closely related to vegetation response. Lefohn and Runeckles (1987) proposed the use of a sigmoidally weighted index for assessing vegetation based on evidence indicating a greater relative importance of higher concentrations in affecting vegetation in comparison to the mid and lower values (Musselman et al., 1983). Lefohn et al. (1988) mathematically described and applied the W126 exposure index to develop exposure-response relationships. The W126 metric is a non-threshold index that is described as the sigmoidally weighted sum of all hourly ozone concentrations observed during a specified daily and seasonal time window, where each hourly ozone concentration is given a weight that increases from zero to one with increasing level. The W126 index has units of ppb-hrs. The W126 exposure index has the form: W126 = $\Sigma\ w_i \times C_i$ with weight $w_i = 1/[1 + M \times exp\ (-A \times C_i/1000)]$, where $M = 4403$, $A = 126$, and where C_i is the hourly average ozone mixing ratio in units of ppb. The M and A constants were derived based on the desire to weight the hourly average levels (1) at a value of one at ≥ 100 ppb and (2) at very low values below 40 ppb. The low weighting at levels below 40 ppb was based on the assumption at the time that hourly average background ozone mixing ratios were mostly associated with levels below this value (US EPA, 2006). As is recognized today, hourly average concentrations associated with background ozone can, at limited times and locations, be significantly higher as a result of stratospheric-tropospheric transport to the surface (Lefohn et al., 2011, 2012, 2014; Emery et al., 2012; Lin et al., 2012; US Federal Register, 2015). For both the vegetation (W126) and human health (W90) sigmoidally weighted exposure indices, the weightings are similar except for the weights at the lower levels (compare Figures 2 and 3).

In the TOAR program, the W126 exposure index is specified over the following time periods: (a) W126 (3-month, 24-h), (b) W126 (6-month, 24-h), (c) W126 (7-month, 24-h), (d) W126 (12-month, 24-h), (e) W126 (3-month, 12-h (0800-1959h), (monthly periods specified)), (f) W126 (6-month, 12-h (0800-1959h), (monthly periods specified)), (g) W126 (7-month, 12-h (0800-1959h), (monthly periods specified)), (h) W126 (12-month, and 12-h (for tropical or subtropical moist climate zones), (0800-1959h)).

The US EPA (US Federal Register, 2015) concluded that ozone effects in plants were cumulative; higher ozone concentrations appeared to be more important than lower concentrations in eliciting a response; plant sensitivity to ozone varied with time of day and plant development stage; and quantifying exposure with indices that cumulate hourly ozone concentrations and preferentially weight the higher concentrations improved the explanatory power of exposure/response models for growth and yield, over using indices based on mean and peak exposure values. As a result of its emphasis on weighting the higher exposures greater than the mid- and low-levels, the US EPA endorsed the use of the W126 exposure index. The Agency concluded that protection of vegetation from adverse effects can be provided by an 8-h ozone standard of 70 ppb that limits cumulative 3-month seasonal W126 exposures to 17 ppm-hrs or lower. The 70 ppb 8-h ozone standard as per the US EPA's 2015 decision (US Federal Register, 2015) serves as a surrogate to achieve ozone levels at or below a W126 value of 17 ppm-hrs. The U.S. EPA applies the three-year average of the annual 4th highest daily 8-h average to determine an exceedance of the standard.

i-r) *AOT40*

AOT40 is the sum of the difference between the hourly mean ozone value at the top of the canopy and values above 40 ppb for all daylight hours over a specified time. In the 1990s, as a response to growing understanding that plants were responding to accumulated ozone above a threshold rather than a long-term average (Fuhrer et al., 1997), the Convention on Long-range Transboundary Air Pollution (CLRTAP) developed and recommended the AOT40 as a threshold-based metric for ozone risk assessment in European plant ecosystems (described in CLRTAP, 2017). In recent years, the CLRTAP has adopted the flux-based metric, POD_Y (see Section 2.3.4) in preference to AOT40 as this metric has greater biological relevance and is better correlated with field evidence of effects (Mills et al., 2011a). It was not feasible to include POD_Y metrics in TOAR. AOT40 is used as legislative standard in Europe (Directive 2008/50/EC), when accumulated over a standard time window (0800-1959 h) and a standard time period (May to July). The CLRTAP has established AOT40-based critical levels for crops, grasslands and forests using vegetation-specific time intervals and AOT40 accumulated during daylight hours. When applied at the global level a variety of accumulation windows are required (see *TOAR-Vegetation* and http://www.igacproject.org/activities/TOAR*)*.

For all the AOT40 TOAR metrics, the following calculation steps were made:

Step 1: Determine the receptor-specific accumulation period.

Step 2: Collate the hourly mean ozone values over the accumulation period.

Step 3: Calculate the AOT40 index by subtracting 40 ppb from each hourly mean value during daylight hours (when global radiation is > 50 W m^{-2}), specific hours

of day (0800 – 1959) or nighttime hours (when global radiation is < 5 W m^{-2}) and then sum the resulting values.

For the purposes of the TOAR database, the AOT40 ozone metric was calculated using the reported hourly ozone values at each site, and no adjustment was made to account for the canopy height of vegetation types. This contrasts with guidelines applied regionally (e.g. in the EU), which adjust to typical canopy heights (CLRTAP, 2017).

i) AOT40 (3-month, 0800-1959 h)

The timing of the two three-month accumulations period for agricultural crops should reflect the period of active growth of wheat and rice, respectively, and be centered on the timing of anthesis, as summarized in *TOAR-Vegetation* and http://www.igacproject.org/activities/TOAR.

The timing of the start of the growing season for horticultural crops is more difficult to define because these crops are repeatedly sown over several months in many regions. For local application, appropriate 3-month periods should be selected.

This metric does not apply to forests.

j) AOT40 (6-month, 0800-1959 h)

This metric typically applies to forests and long-lived perennial vegetation, such as grasslands. The default exposure windows for the accumulation are suggested in *TOAR Vegetation*. These time periods do not take altitudinal variation into account, should be viewed as indicative only, and should only be used where local information is not available.

k) AOT40 (7-month, 0800-1959 h)

For consistency with other metrics (e.g., W126), a 7-month accumulation period was recommended for inclusion in TOAR.

l) AOT40 (12-month, 0800-1959 h)

This metric is calculated as stated above, but the accumulation period is the entire year. It applies to species with a year-long growing season (e.g., Mediterranean evergreen forests, tropical and subtropical moist climate forests).

m) AOT40 (3-month, daylight over the period when clear sky radiation > 50 W m^{-2}*)*

This metric is calculated as i), but rather than being accumulated over the hours from 8:00 to 19:59, it is restricted to those hours when clear sky radiation exceeds 50 W/m^2. This value is recommended by CLRTAP (2017) to ensure that stomata are open (daylight) and ozone in the air is absorbed within the leaf. Radiation

values in TOAR are estimated by modeling according to Blanco-Muriel et al. (2001), where "irradiance > 50 W/m^2" is similar to the condition "elevation angle > 5 degrees". The threshold for daylight versus night is 5 degrees solar elevation angle, which is used as surrogate for 50 W m^{-2} (see *TOAR-Surface Ozone Database*).

n) AOT40 (6-month, daylight over the period when clear sky radiation > 50 W m^{-2}*)*

This metric is calculated as j) and with the same rationale as m).

o) AOT40 (7-month, daylight over the period when clear sky radiation > 50 W m^{-2}*)*

This metric is calculated as k) and with the same rationale as m).

p) AOT40 (3-month, nighttime over the period when clear sky radiation < 5 W m^{-2}*)*

This metric is calculated as m), but only includes all the nighttime hours, when stomata should be closed. It is restricted to those hours when clear sky radiation is below 5 W/m^2. Although a 0 threshold would be more biologically-based, an arbitrary 5 threshold ensures that only nighttime values are included and can be easily handled in a large database. Radiation values are again estimated according to Blanco-Muriel et al. (2001), where "irradiance < 5 W m^{-2}" is similar to the condition "elevation angle < -5 degrees", and leaves out dawn and dusk hours entirely.

q) AOT40 (6-month, nighttime over the period when clear sky radiation < 5 W m^{-2}*).*

This metric is calculated as n) and with the same rationale as p).

r) AOT40 (7-month, nighttime over the period when clear sky radiation < 5 W m^{-2}*).*

This metric is calculated as o) and with the same rationale as p).

s-v) Daily 12-h Average

Besides AOT40, the daily 12-h (0800-1959h mean ozone exposure metric) (M12) has been widely used to characterize crop exposures to establish crop-specific exposure–response relationships, which relate a quantifiable mean to a reduction in crop yield (Heck et al., 1988; Jäger et al., 1992; Legge et al., 1995; Van Dingenen et al., 2009). These resulted in the derivation of robust exposure–response relationships for a number of key agricultural crops. Notably, the growing season time window on which M12 exposure response relationships are based depend on vegetation types and climate zones. However, in post-experimental data analysis, cumulative metrics, such as the SUM06 and W126 indices (US EPA, 2013; US Federal Register, 2015) better fit the yield loss observations for experiments conducted in the US, and thus received greater focus (Tingey et al., 1991; Lefohn and Foley, 1992; Mauzerall & Wang, 2001). As a

result of this, fewer studies use the M12 as an exposure metric for assessing crop or tree injury and damage.

s) Daily 12-h average averaged over 3 months, (0800-1959h)

Similarly to AOT40, this metric is relevant for agricultural crops and the same timings of the representative growing seasons for wheat and rice summarized in *TOAR-Vegetation* and http://www.igacproject.org/activities/TOARwere used to derive this metric.

(t) Daily 12-h average averaged over 6 months, (0800-1959h) (monthly periods specified in accompanying documentation)

This is typical time window for deciduous forests and semi-natural vegetation. The default exposure windows for the accumulation are shown in *TOAR-Vegetation* and http://www.igacproject.org/activities/TOAR. This metrics does not apply to the forest whose growth period during the year is less than 6 months (e.g. boreal forest).

(u) Daily 12-h average averaged over 7 months, (0800-1959h) (monthly periods specified in accompanying documentation).

This is a typical time window for deciduous forests and semi-natural vegetation in temperate moist or subtropical climate zones. The default exposure windows for the accumulation are shown in *TOAR-Vegetation* and http://www.igacproject.org/activities/TOAR. This metrics does not apply to forests whose growth period during the year is less than 7 months (e.g. boreal forest).

(v) Daily 12-h average averaged over 12 months, (0800-1959h).

This is the typical time window for evergreen forests and semi-natural vegetation in Mediterranean and sub-tropical climate zones.

w) Stomatal flux

In Europe, since the late 1990s, it has been recognized by the CLRTAP that ozone effects on vegetation are related to the uptake of ozone through the stomatal pores of plants. The DO_3SE model (Emberson et al., 2000) was adopted by CLRTAP for calculating the accumulated stomatal flux of ozone from hourly values of ozone level, together with conductance modifying factors: temperature, vapor pressure deficit, light (irradiance), soil water potential (SWP) or plant available water (PAW), ozone value and plant development stage (phenology). For this metric, the hourly mean instantaneous stomatal flux of ozone based on the projected leaf area (PLA), F_{st} (in nmol m^{-2} PLA s^{-1}), is accumulated over a stomatal flux threshold of Y nmol m^{-2} s^{-1}. The accumulated Phytotoxic Ozone Dose (i.e., the accumulated stomatal flux) of ozone above a flux

threshold of Y (POD_Y, formerly named $AF_{st}Y$), is calculated for the appropriate time-window as the sum over time of the differences between hourly mean values of F_{st} and Y nmol m^{-2} PLA s^{-1} for the periods when F_{st} exceeds Y. The Y threshold varies between species as do the parameterizations for each flux modifying factor such as temperature or soil moisture, reflecting the different stomatal dynamics of different species. Two types of POD_Y model exist: POD_YIAM which has a simplified parameterization and is suitable for large-scale integrated assessment, and POD_YSPEC, species-specific parameterization of the flux model. Local and regional parameterizations have been defined for POD_YSPEC and POD_YIAM in CLRTAP (2017) for a range of crops, tree and grassland species/species groups and used to define 21 critical levels, above which negative effects of ozone on crop yield, biodiversity and tree growth are expected (CLRTAP, 2017). Further information can be found in summary in Mills et al. (2011b), and in more detail including response functions in Grünhage et al. (2012) for wheat, Gonzalez et al. (2014) for tomato, and Büker et al. (2015) for tree species. The POD_Y model has also been applied in China to derive flux-effect relationships for wheat (Feng et al., 2012) and poplar (Hu et al., 2015).

x) Seasonal Percentiles (median, 5th, 25th, 75th, 95th, 98th, and 99th).

The magnitude of each exposure and dose metric is influenced by the hourly average concentrations occurring within the ozone distribution. As outlined in Sections 1 and 2, the changes in the magnitude and trend pattern for exposure indices relevant for both vegetation and human health are determined by the relationship between each metric and changes that occur in the different parts of the ozone distribution. The shifts that occur among the hourly average levels can occur because of changes (increases or reduction) in the extent of NO_x titration, photochemical ozone production, background ozone levels, and ozone deposition (Lefohn and Cooper, 2015; Monks et al., 2015). The seasonal 5th, 25th, 50th, 75th, 95th, 98th, and 99th percentile values of hourly ozone levels are of interest so that each of the specific percentiles can be trended on a seasonal basis. This information is very helpful in relating the changes which occur in the exposure and dose metrics to the changes occurring across the ozone distribution in different seasons.

Table S-4: Percentage of sites included in TOAR for which the trends (from 1995 through 2014) in the TOAR exposure metrics (columns) were in the same direction (i.e., decreasing, increasing, or no significant change) compared to a set of metrics (rows), which included those in Lefohn et al. (2017).

	4th W90	4th dma8epa	SOMO35	SOMO10	3-month aot40 wheat	6-month aot40 summer	3-month w126_12h wheat	6-month w126_12h summer	3-month w126_24h wheat	6-month w126_24h summer	3-month M12 wheat	6-month M12 summer
**4th W90**	100%	92%	67%	53%	57%	75%	62%	80%	60%	77%	46%	57%
**4th dma8epa**	92%	100%	69%	56%	61%	78%	66%	81%	63%	79%	48%	60%
**SOMO35**	67%	69%	100%	81%	80%	85%	79%	81%	79%	83%	67%	82%
**SOMO10**	53%	56%	81%	100%	78%	70%	75%	65%	75%	67%	76%	84%
**3-month aot40 wheat**	57%	61%	80%	78%	100%	74%	89%	67%	91%	69%	80%	77%
**6-month aot40 summer**	75%	78%	85%	70%	74%	100%	78%	92%	76%	92%	58%	78%
**3-month w126_12h wheat**	62%	66%	79%	75%	89%	78%	100%	73%	94%	74%	72%	76%
**6-month w126_12h summer**	80%	81%	81%	65%	67%	92%	73%	100%	71%	95%	52%	72%
**3-month w126_24h wheat**	60%	63%	79%	75%	91%	76%	94%	71%	100%	73%	74%	75%
**6-month w126_24h summer**	77%	79%	83%	67%	69%	92%	74%	95%	73%	100%	54%	74%
**3-month M12 wheat**	46%	48%	67%	76%	80%	58%	72%	52%	74%	54%	100%	72%
**6-month M12 summer**	57%	60%	82%	84%	77%	78%	76%	72%	75%	74%	72%	100%

* For two of the TOAR exposure metrics (i.e., AmaxMDA8 and AmaxMDA1) analyzed in Lefohn et al. (2017), trend analyses were not available in the TOAR preformatted files.

Additional References Cited in Supplement Not Listed in the Main Paper

Anenberg, SC, Horowitz, LW, Tong, DQ, West, JJ. 2010. An estimate of the global burden of anthropogenic ozone and fine particulate matter on premature human mortality using atmospheric modeling. *Environ Health Persp*, 118, 1189-1195. doi:10.1289/ehp.0901220.

Atkinson RW, Yu D, Armstrong BG, Pattenden S, Wilkinson P, et al. 2012. Concentration-response function for ozone and daily mortality: Results from five urban and five rural UK populations. *Environ Health Perspect* **120**(10): 1411-1417.

Bell ML, Peng RD, Dominici F. 2006. The exposure-response curve for ozone and risk of mortality and the adequacy of current ozone regulations. *Environ Health Persp* **114**(4): 532-536.

Brauer M, Markus A, Burnett RT, Cohen A, Dentener F, et al. 2012. Exposure assessment for estimation of the global burden of disease attributable to outdoor air pollution. *Environ Sci & Tec*h **46** (2): 652–60. doi:10.1021/es2025752.

Chen R, Cai J, Meng X, Kim H, Honda Y, et al. 2014. Ozone and daily mortality rate in 21 cities of East Asia: How does season modify the association? *Amer J of Epidem* **180**(7): 729-736. doi:10.1093/aje/kwu183.

Emery C, Jung J, Downey N, Johnson J, Jimenez M, et al. 2012. Regional and global modeling estimates of policy relevant background ozone over the United States. *Atmos Environ* **47**: 206-217.

EPA Victoria. 2014. Air monitoring report 2013 - compliance with the National Environment Protection (Ambient Air Quality) Measure. EPA Victoria Publication number 1569. Environmental Protection Agency Victoria. Available at: http://www.epa.vic.gov.au/~/media/Publications/1569.pdf (accessed on 18 October 2017).

Garber W, Colosio J, Grittner S, Larssen S, Rasse D, et al. 2002. Guidance on the Annexes to Decision 97/101/EC on Exchange of Information as revised by Decision 2001/752/EC. Available at: http://ec.europa.eu/environment/air/pdf/guidancetoannexes97101ec.pdf (accessed on 18 October 2017).

Kickert RN, Krupa SV. 1991. Modeling plant response to tropospheric ozone: A critical review. *Environ Pollut* **70**(4): 271 383.

Kim SY, Lee JT, Hong YC, Ahn KJ, Kim H. 2004. Determining the threshold effect of ozone on daily mortality: an analysis of ozone and mortality in Seoul, Korea, 1995-1999. *Environ Res* **94**(2): 113-119.

Lefohn AS, Cooper OR. 2015. Introduction to the special issue on observations and source attribution of ozone in rural regions of the western United States. *Atmos Environ* **109**: 279-281. doi: 10.1016/j.atmosenv.2015.03.030.

Lefohn AS, Emery C, Shadwick D, Wernli H, Jung J, et al. 2014. Estimates of background surface ozone concentrations in the United States based on model-derived source apportionment. *Atmos Environ* **84**: 275-288. doi: 10.1016/j.atmosenv.2013.11.033.

Lefohn AS, Wernli H, Shadwick D, Limbach S, Oltmans S, et al. 2011. The importance of stratospheric-tropospheric transport in affecting surface ozone concentrations in the Western and Northern Tier of the United States. *Atmos Environ* **45**: 4845-4857.

Lefohn AS, Wernli H, Shadwick D, Oltmans SJ, Shapiro M. 2012. Quantifying the frequency of stratospheric-tropospheric transport affecting enhanced surface ozone concentrations at high- and low-elevation monitoring sites in the United States. *Atmos Environ* **62**: 646-656.

Lim SS, Vos T, Flaxman AD, Danaei G, Shibuya K, et al. 2012. A comparative risk assessment of burden of disease and injury attributable to 67 risk factors and risk factor clusters in 21 regions, 1990-2010: A systematic analysis for the global burden of disease study 2010. *Lancet* **380** (9859): 2224–60. doi:10.1016/S0140-6736(12)61766-8.

Lin M, Fiore AM, Cooper OR, Horowitz LW, Langford AO, et al. 2012. Springtime high surface ozone events over the western United States: Quantifying the role of stratospheric intrusions. *J Geophy Res* **117**: D00V22, doi:10.1029/2012JD018151.

Massman WJ, Musselman RC, Lefohn AS. 2000. A conceptual ozone dose-response model to develop a standard to protect vegetation. *Atmos Environ* **34(5):** 745-759.

Ministry of Environmental Protection of the People's Republic of China. 2012. Ambient Air Quality Standards, GB3095-2012.

Rombout PJA, Lioy PJ, Goldstein BD. 1986. Rationale for an eight-hour ozone standard. *JAPCA* **36(8):** 913-916.

Royal Society. 2008. Ground-level ozone in the 21st century: future trends, impacts and policy implications. London: The Royal Society. Science Policy, 15/08. Available at: http://royalsociety.org/uploadedFiles/Royal_Society_Content/policy/publications/2008/7925.pdf (accessed on 18 October 2017).

Schelegle ES, Morales CA, Walby WF, Marion S, Allen RP. 2009. 6.6-hour inhalation of ozone concentrations from 60 to 87 ppb in healthy humans, *Am J Respir Crit Care Med* **180**: 265–272.

Shindell DT, Lee Y, Faluvegi G. 2016. Climate and health impacts of US emissions reductions consistent with 2 degrees, C. *Nature Climate Change*, **6**: 503-507. doi:10.1038/nclimate2935.

US EPA. 2006. Air Quality Criteria for Ozone and Related Photochemical Oxidants (2006 Final). EPA/600/R-05/004aF-cF. Washington, DC: Environmental Protection Agency. Available at: http://cfpub.epa.gov/ncea/cfm/recordisplay.cfm?deid=149923 (accessed on 18 October 2017).

US Federal Register. 1997. National Ambient Air Quality Standards for Ozone, 62, No. 138, 40 CFR Part 50, 38856-38896.

Van Dingenen R, Dentener FJ, Raes F, Krol MC, Emberson L, et al. 2009. The global impact of ozone on agricultural crop yields under current and future air quality legislation. *Atmos Environ* **43**: 604-618.
